# Multi-cohort ensemble learning framework for vaginal microbiome-based endometrial cancer detection

**DOI:** 10.3389/fcimb.2025.1641413

**Published:** 2025-12-08

**Authors:** Dollina Dodani, Aline Talhouk

**Affiliations:** Department of Obstetrics and Gynecology, Division of Gynecologic Oncology, University of British Columbia, Vancouver, BC, Canada

**Keywords:** endometrial cancer, 16S rRNA, machine learning, data integration, biomarkers, reproducibility, vaginal microbiome, multi-cohort analysis

## Abstract

**Introduction:**

Endometrial cancer is the most common gynecological malignancy in high-income countries and lacks an established strategy for early detection. Prior studies suggest that the vaginal microbiome may hold diagnostic potential, but inconsistent findings have limited clinical translation.

**Methods:**

We conducted a systematic review to collect and analyze vaginal 16S rRNA sequencing data from five independent cohorts (n = 265). These studies included women with histologically confirmed endometrial cancer and controls with benign gynecologic conditions. We used these datasets to identify microbial signatures associated with endometrial cancer and to develop a predictive machine learning model.

**Results:**

Microbial diversity was significantly higher in endometrial cancer samples, and host characteristics influenced community composition. *Peptoniphilus* was reproducibly enriched in cancer samples across cohorts. An ensemble classifier accurately identified endometrial cancer in a held-out test set, achieving an area under the receiver operating characteristic curve of 0.93 (95% CI: 0.71–0.93), sensitivity of 1.0 (95% CI: 0.74–1.0), and a negative predictive value of 1.0 (95% CI: 0.59–1.0).

**Discussion:**

These findings support the potential of vaginal microbiome profiling as a minimally invasive approach for early detection of endometrial cancer.

## 1 Introduction

Endometrial cancer (EC) is the most common gynecological malignancy in high-income countries, with incidence rising globally (Bray et al., 2024; Siegel et al., 2024), due to increasing obesity rates, sedentary lifestyles, and aging populations (Reeves et al., 2007; Onstad et al., 2016). When diagnosed while still confined to the uterus, EC is treatable with hysterectomy, with a 5-year survival rate exceeding 90% (Uterine cancer survival statistics, 2015). However, survival drops to 17% in metastatic disease, underscoring the need for earlier detection (Uterine cancer survival statistics, 2015).

Abnormal uterine bleeding (AUB) is the most common first presenting symptom and prompts diagnostic endometrial biopsy, the current gold standard (Qureshi et al., 2018). While 90% of women diagnosed with EC report AUB, this common symptom during perimenopause lacks specificity, with fewer than 1% of pre-menopausal and 9% of post-menopausal women with AUB diagnosed with EC (Iram et al., 2010; CLARKE et al., 2020). Moreover, endometrial biopsies are invasive and painful (Marcus et al., 2021). There is a critical need for minimally invasive tests that can rule out malignancy (Costas et al., 2019).

Advances in next-generation sequencing and fluid-based sampling techniques have accelerated microbiome research, opening opportunities for minimally invasive biomarker-based cancer screening (Łaniewski et al., 2020; Chambers et al., 2021). Unlike gut microbial signatures that have led to early detection of colon cancer (Fusco et al., 2024), clinical translation of vaginal microbiome signatures remains limited. Several studies using 16S rRNA gene sequencing have identified associations between vaginal bacterial composition and EC, but reproducibility across datasets remains a challenge. Small sample sizes, inter-individual microbiome variability, and inconsistent bioinformatics pipelines contribute to varying results. Cross-study comparisons of bioinformatics pipelines have improved reproducibility in oral and gut microbiomics, but this type of comparison has not yet been conducted for the vaginal microbiome (Kang et al., 2021; Fox et al., 2024). Additionally, machine learning techniques can be employed to integrate data from multiple cohorts to identify a predictive vaginal microbiome signature.

To address this, we conducted a multicohort analysis of publicly available 16S rRNA gene vaginal microbiome datasets from EC case-control studies. We systematically evaluated and selected a bioinformatics pipeline based on its reproducibility and replicability following the framework proposed by Hejblum et al., 2020. We also evaluated an additional pipeline that uses recent microbiome processing advancements. The resulting microbiome profiles were used to train a machine-learning classifier that incorporates individual characteristics, such as age, body mass index (BMI), and ethnicity, which influence both the vaginal microbiome and the risk of developing EC and its precursor, atypical endometrial hyperplasia (AEH).

## 2 Methods

### 2.1 Dataset search & inclusion

We used PubMed to systematically search for studies that analyzed 16S rRNA amplicon sequence microbiome data from the vaginal microbiome in individuals with EC and control groups (see **Supplementary Section 1** for search keywords). To reduce variability, we included only those studies that collected specimens *via* vaginal swabs or uterine lavages, used 16S rRNA gene sequencing (any region), and provided publicly available raw sequence data with pathology labels. We excluded studies that used metagenomics, non-amplicon markers, or sampled endometrial tissue. For studies with longitudinal sampling or multiple anatomical sites, only baseline (cervico) vaginal samples were included. Atypical endometrial hyperplasia was grouped with EC, because they are likely to arise together and are clinically treated similarly. All other pathologies, including simple hyperplasia, were deemed benign. Sequence data and metadata were retrieved from the Short Read Archive or materials provided by study authors.

### 2.2 Selection of bioinformatics methods

Our goal was to identify a bioinformatics pipeline that consistently reproduced and replicated key microbiome metrics across various studies, including 1) Alpha diversity, measuring diversity within samples, 2) Beta diversity, to measure clustering of microbial communities based on their composition, and 3) differentially abundant taxa associated with disease status (Douglas and Langille, 2021). We define reproducibility as obtaining similar results using the original authors’ analysis pipeline on their dataset and replicability as achieving comparable results when applying that same pipeline to a different dataset (Hejblum et al., 2020). We extracted bioinformatics workflows from published manuscripts.

Each dataset was reanalyzed using both the original pipeline, the pipelines from other published studies, as well as the DADA2 pipeline, a high-resolution denoiser suitable for identifying rare taxa, particularly relevant in *Lactobacillus*-dominated vaginal communities (Callahan et al., 2016; Nearing et al., 2018). Samples were excluded based on pipeline-specific quality thresholds or incomplete metadata (**Supplementary Section 2**). Processing steps for the DADA2 pipeline included, adapter trimming using BBDuk^1^, quality filtering using Phred scores and trimming the 3’ ends of reads where the average quality dropped below 20 (see **Supplementary Section 3** for read lengths maintained). Forward and reverse reads were denoised and paired-end reads were merged. In studies where there was minimum to no read overlap (<50% samples merging with >12bp overlap; details in **Supplementary Section 3**), only forward reads were used as previously done by (Sekaran et al., 2023; Abdill et al., 2025). After filtering out bimeras, the Amplicon Sequence Variants (ASVs) abundance table was normalized by the total number of reads sequenced in each sample. Identified ASVs were assigned species-level taxonomic information using the naïve Bayes classifier implemented in QIIME2 based on 1) the Green Genes (GG) database (v13.8), 2) the Genome Taxonomic Database (GTDB) (vbac120) both with uniform taxonomic distribution, and 3) the GTDB database with an expected species distribution using q2-clawback (DeSantis et al., 2006; Fettweis et al., 2012; Bokulich et al., 2018, Bokulich et al., 2022; Bolyen et al., 2019; Kaehler et al., 2019; Rinke et al., 2021).

To merge taxonomies from GG and GTDB, we used superstring matching and RESCRIPt to generate a consensus taxonomy based on the last common ancestor (Robeson et al., 2021; Bokulich et al., 2022). A phylogenetic tree was built by aligning ASVs with MAFFT, processed with FastTree, and midpoint-rooted using the phangorn package (v2.11.1) in R (Price et al., 2010; Katoh and Standley, 2013). **Supplementary Section 4** outlines the bioinformatics pipelines implemented.

For each dataset, we compared the performance of the previously implemented pipelines with DADA2. We used the Shannon index to measure alpha diversity. For beta diversity, we tested the marginal significance of available participant characteristics and disease status using the PERMANOVA test (did not implement multiple testing correction), along with distance measures reported in individual studies (such as Bray-Curtis, calculated using the relative abundances of taxonomic features, and (weighted or unweighted) UniFrac, which considers the phylogeny of the taxonomic features as well) (Bray and Curtis, 1957; Lozupone et al., 2011; Anderson, 2017). For the DADA2 pipeline, we used the Bray-Curtis, Jaccard (Real and Vargas, 1996), UniFrac, and Jensen-Shannon Divergence (Fuglede and Topsoe, 2004) metrics. The weighted UniFrac metric was calculated using the phylogenetic tree generated from ASVs. Both alpha and beta diversity were measured at the ASV level. To identify differentially abundant taxa in EC participants, we aggregated the abundance tables to the species level (or the genus taxonomic level, if unassigned at the species level) and used ANCOM-BC with multiple testing adjustment using the Holm method (Lin and Peddada, 2020). All microbiome metrics evaluated were adjusted for potential confounding patient factors, including age, BMI, and ethnicity. The pipeline that consistently demonstrated trends reported in literature for alpha/beta diversity and associated EC taxa was selected for downstream predictive modeling.

### 2.3 Data integration, model development, and selection of validation cohort

**Figure 1:**
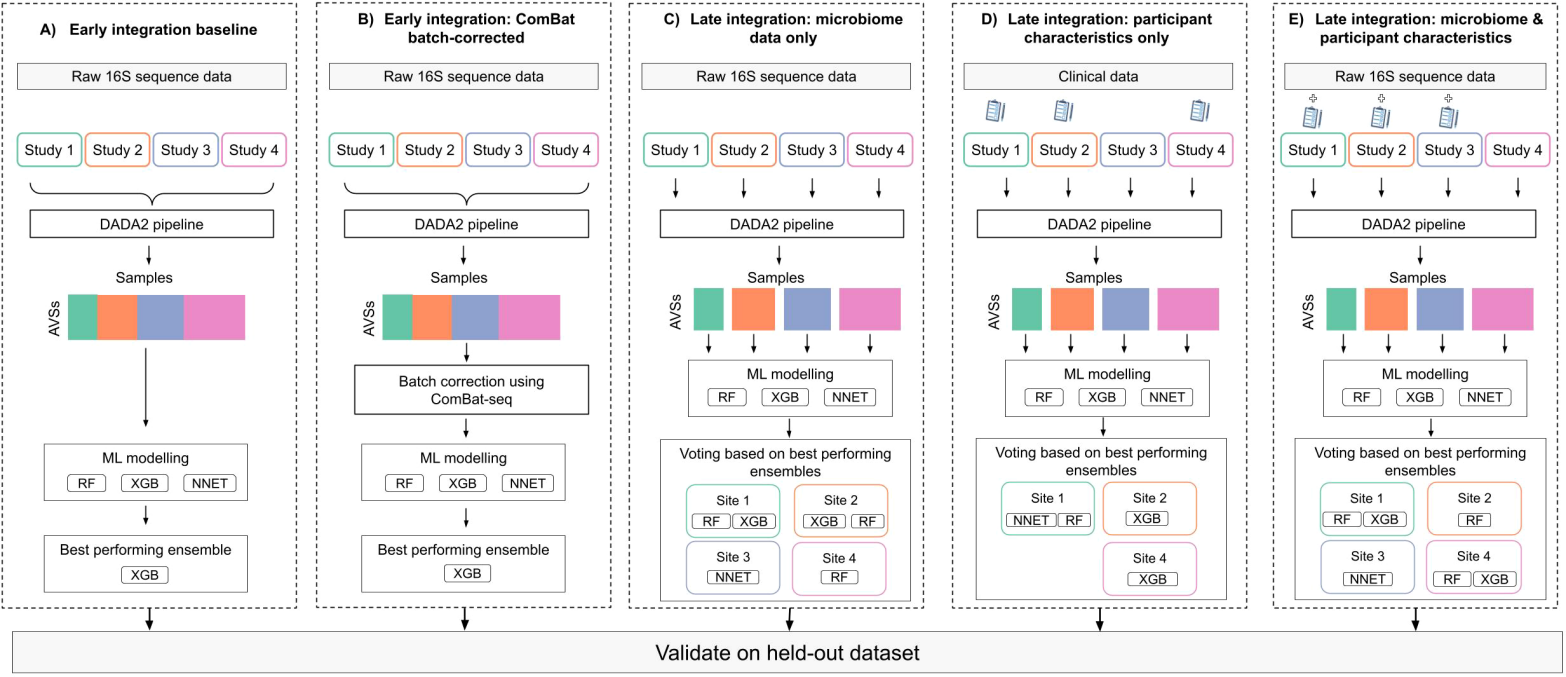
Depiction of modelling frameworks implementing different integration strategies: 1) Early integration where all datasets are aggregated into a single data frame prior to modelling with **(A)** Non-batch corrected and **(B)** ComBat batch-corrected data. 2) Late integration where a local model is trained on each study data and final predictions are averaged across all studies. Models built using **(C)** microbiome data only, **(D)** patient characteristics only, and **(E)** both microbiome and patient characteristic data. The best-performing ensemble was selected based on internal validation (out of fold) error and evaluated on the held-out dataset.

We compared several data integration strategies to develop a predictive vaginal microbiome signature for EC (**Figure 1**). One study, Antonio et al (Walther-António et al., 2016). was set aside for validation. This was selected because it was not too large nor too small and had balanced number of EC and benign diagnoses. The remaining studies were used for model training. Our *baseline approach* used early integration, where datasets were concatenated into a single high-dimensional matrix (Picard et al., 2021). In the *batch-corrected* approach, we used ComBat Johnson et al., 2007) to adjust for batch effects and assessed clustering by batch versus disease status using the PERMANOVA test (Anderson, 2017).

We also evaluate *a late integration* strategy, where separate classifiers were trained on individual datasets and combined to generate ensemble predictions (Mienye and Sun, 2022). This allowed the inclusion of patient-level characteristics when available and permitted the use of different algorithms for different datasets (Mienye and Sun, 2022).

Under late integration, we trained four models; (1) a microbiome-only model, (2) a model using patient characteristics (age, BMI, and ethnicity) only, (3) a model combining patient characteristics and microbiome data, (4) a model using patient characteristics and vaginal pH as a microbiome biomarker, since vaginal pH is a consequence of the vaginal microbiome composition and associated with EC risk (Walther-António et al., 2016).

All models were built with CLR transformed, genus-level count data and adhering to the Transparent Reporting of a multivariable prediction model for Individual Prognosis Or Diagnosis + Artificial Intelligence guidelines (Collins et al., 2024) (checklist in **Supplementary Section 5**). Taxa present in fewer than 5% of samples were filtered out. For each integration strategy, we evaluated the following algorithms: random forest algorithm (randomForest v4.7-1.1), gradient tree boosting algorithm (xgboost v1.7.7.1), and neural network algorithm (nnet v7.3-19) using the tidymodels (v1.2.0) package. We also evaluated ensembles comprising three base models. Hyperparameter tuning was performed using grid search (details in **Supplementary Section 6**) with a five-fold stratified cross-validation, repeated three times, and optimized for F1 score at a 0.5 classification threshold. Final ensemble models were selected based on out-of-fold performance and tested on a held-out test. To demonstrate the generalizability of the model that performs the best on the held-out dataset, we implemented a leave-one-study-out (LOSO) validation approach, retraining the models each time to only include the covariates available in the held-out dataset.

We reported sensitivity, specificity, negative predictive value (NPV), positive predictive value (PPV), and area under the receiver operating characteristics (AUROC) using the yardstick (v1.3.1) package. For the LOSO validation, we report pooled metrics. Exact 95% confidence intervals were calculated using epiR (v2.0.80). To correct for class imbalance, we used SMOTENC from the themis package (v1.0.3). For datasets with partially missing data, Multivariate Imputation by Chained Equations (mice v3.16.0) was used to impute missing values, conditioned on other participant data and microbiome profile. All analyses were performed in R (v4.3.3) and RStudio (v2023.06.2). Executable code and pipeline parameters are available on Github^2^.

## 3 Results

### 3.1 Datasets & bioinformatics pipelines

The systematic literature review yielded 248 articles, of which 11 used 16S rRNA amplicon sequencing to assess vaginal microbiome profiles. We excluded 5 studies that relied on invasive tissue sampling and 1 study that did not provide access to sequencing data, resulting in 5 eligible datasets for analysis.

The first study, published by the Mayo Clinic in 2016, included 22 participants and investigated the vaginal microbiome composition and its putative role in EC (Walther-António et al., 2016). The study reported significant age differences between EC patients and controls with benign conditions (mean age 62 vs 47 years). A follow-up study by the same group in 2019 expanded the cohort to 149 participants and evaluated participant characteristics, including menopausal status, BMI, vaginal pH, and age, and their associations with microbial composition (Walsh et al., 2019). This second cohort demonstrated differences in EC patients compared to those with benign diagnoses. Notably, EC patients were older, had higher BMI and vaginal pH (**Table 1**).

**Table 1:** Patient and study characteristics in included cohorts.

Study (Year)	16S rRNA region	Benign (N = 130)	EC (N = 135)	Total (N = 265)	p-value
Walther-António et al., 2016	V3-V5	10	12	22	
Age, mean ± SD		47.1 ± 9.0	62.3 ± 8.7	63.6 ± 8.6	**< 0.05**
BMI, mean ± SD		29.2 ± 6.89	34.8 ± 7.7	35.4 ± 8.1	0.08
Vaginal pH					0.17
<= 4.5 (%)		5 (50%)	2 (16.7%)	7 (31.8%)	
> 4.5 (%)		5 (50%)	9 (75%)	14 (63.6%)	
NA (%)			1 (8.3%)	1 (4.5%)	
Ethnicity					
White (%)		10 (100%)	12 (100%)	22 (100%)	
Walsh et al., 2019	V3-V5	80	69	149	
Age, mean ± SD		50.4 ± 10.2	61.3 ± 10.8	55.4 ± 11.5	**< 0.05**
BMI, mean ± SD		31.7 ± 9.0	35.6 ± 9.6	33.7 ± 9.4	**< 0.05**
Vaginal pH					**< 0.05**
<= 4.5 (%)		25 (31.3%)	3 (4.3%)	28 (18.8%)	
> 4.5 (%)		47 (58.8%)	55 (79.7%)	102 (68.5%)	
NA (%)		8 (10%)	11 (15.9%)	19 (12.8%)	
Ethnicity					0.06
White (%)		59 (73.8%)	60 (87%)	110 (73.8%)	
Other (%)		21 (26.3%)	9 (13%)	30 (20.1%)	
Tsementzi et al, 2020	V4	28	8	36	
Age, mean ± SD		63.7 ± 6.4	61.1 ± 5.4	63.5 ± 6.3	
BMI, mean ± SD		27.1 ± 6.5	32.0 ± 8.4	26.9 ± 6.5	
Vaginal pH					**< 0.05**
<= 4.5 (%)		4 (14.3%)	1 (12.5%)	5 (13.9%)	
> 4.5 (%)		21 (75%)	3 (37.5%)	24 (66.7%)	
NA (%)		3 (10.7%)	4 (50%)	7 (19.4%)	
Ethnicity					0.11
White (%)		17 (60.7%)	2 (25%)	19 (52.8%)	
Other (%)		11 (39.3%)	6 (75%)	17 (47.2%)	
Gressel et al, 2021	V4	4	23	27	
Chao et al., 2022	V3-V4	8	23	31	
Age, mean ± SD		44.6 ± 12.2	44.4 ± 11.1	43.3 ± 10.5	0.77

Significant differences (p < 0.05) are indicated in bold.

Tsementzi et al., 2020 analyzed vaginal microbiome composition in EC and cervical cancer (n=38) compared to participants with benign conditions. EC patients had significantly higher vaginal pH (> 4.5) compared to controls. Gressel et al., 2021, examined postmenopausal women (n=27), profiling cervicovaginal microbiota to identify taxa associated with EC. Most recently, Chao et al., 2022 used lavage-based vaginal sampling to compare microbial taxa associated with EC and benign diagnoses (n=31). Patient characteristics (age, BMI, ethnicity, and vaginal pH) were available for all cohorts except Chao et al. (age only) and Gressel et al. (no patient metadata). The available demographic and clinical characteristics of the participants are summarized in **Table 1**.

Biological specimens across all studies were collected by physicians; all cohorts were recruited in the United States of America except for Chao et al., which enrolled participants in China. Genomic DNA extraction protocol varied: the Mayo Clinic studies used the MoBio PowerSoil Kit, Tsementzi et al. used the DNeasy PowerSoil Kit, and Gressel and Chao et al. used the QIAamp DNA Mini Kit. All studies sequenced the 16S rRNA gene on the Illumina MiSeq platform, targeting various hypervariable regions, with V4 being the common region across all datasets. Study-specific details, including inclusion/exclusion criteria, sampling and storage protocols, DNA extraction, primer design, and controls, are provided in **Supplementary Section 7**.

Bioinformatic pipelines also varied across studies: Antonio et al. and Walsh et al. employed IM-TORNADO (Jeraldo et al., 2014), an in-house pipeline that concatenates paired end-reads and processes them using Mothur and USEARCH to generate Operational Taxonomic Units (OTUs) (Schloss et al., 2009; Edgar, 2010). Chao et al. used UNOISE to denoise sequences and produce ASVs (Edgar and Flyvbjerg, 2015). Tsementzi et al. Gressel et al. used QIIME2 with VSEARCH and USEARCH, respectively, to generate OTUs (Edgar, 2010; Rognes et al., 2016; Bolyen et al., 2019). We reprocessed all the datasets using those four pipelines and added a pipeline based on DADA2 (a total of 25 data/pipeline combinations).

### 3.2 Microbial diversity in various conditions

**Figure 2:**
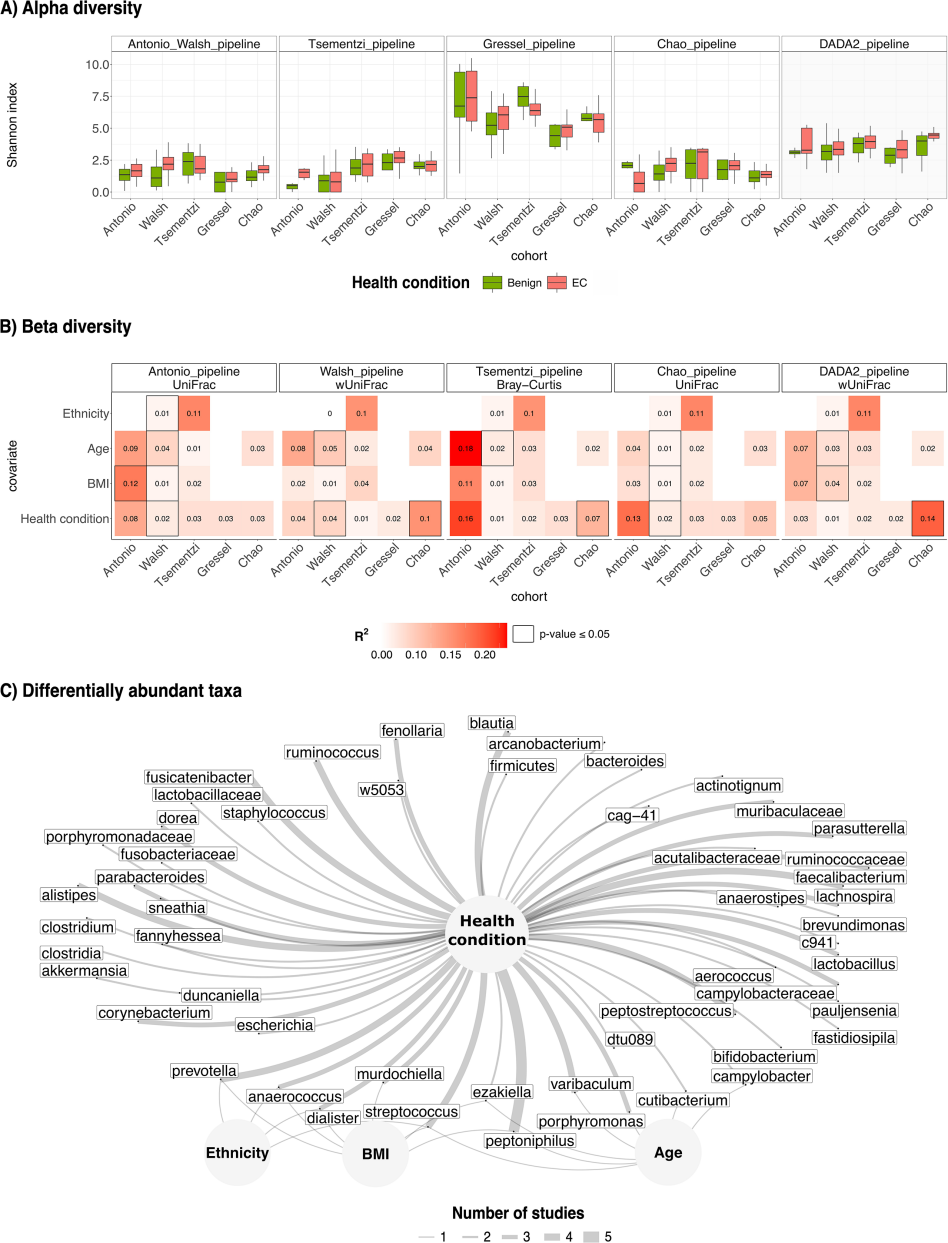
Reproducibility and replicability of alpha, beta diversity and differentially abundant taxa. **(A)** Each panel provides the Shannon index (y-axis) calculated across the two health conditions on each cohort (x-axis) after processing by the pipeline in the respective panel. The last panel is the DADA2 pipeline; the only pipeline that demonstrated higher Shannon index in EC in comparison to benign across all datasets. **(B)** Each panel represents the marginal proportion of variance explained by participant characteristics (y-axis) available in each dataset when (x-axis) processed by the pipeline in the respective panel. Consistent trends were observed across pipelines. Health conditions explain less than 16% of variance in all datasets, whereas individual characteristics appear to have more influence on the structure and composition of the vaginal microbiome.**(C)** Illustration of differentially abundant taxa (using ANCOM-BC) while accounting participant characteristics (nodes in the network plot) where available. The thickness of the edges represents the number of studies that were found to have a multivariate association between two nodes. Various species of *Peptoniphilus* were differentially abundant in EC individuals in all studies.

All included studies originally reported increased vaginal microbial (alpha) diversity in EC patients compared to controls. We were able to reproduce this trend across datasets; however, results varied depending on the bioinformatics pipeline used (**Figure 2A**). The expected trend was not observed in the Tsementzi cohort when processed using the IM-TORNADO pipeline, nor in the Walsh cohort processed with the Tsementzi pipeline. Similarly, alpha diversity was not recapitulated in the Tsementzi and Chao datasets using the Gressel pipeline or in the Antonio dataset using the Chao pipeline. Among all datasets, the Gressel cohort demonstrated the most consistent trends across pipelines and the best quality profile (see **Supplementary Section 3**). However, their pipeline, which does not filter for chimeric reads, systematically produced inflated alpha diversity values in other datasets.

In contrast, processing with DADA2 consistently replicated the expected alpha diversity trend across all datasets (with no significant differences observed), suggesting greater robustness to technical variability.

### 3.3 Intra-variability vs inter-variability

Beta diversity appeared to be less sensitive to preprocessing differences (**Figure 2B**). Biological trends remained largely consistent across pipelines and metrics. We note that disease status accounted for less than 16% of the variance in all cohorts, regardless of the pipeline or distance metric (see **Supplementary Section 8**). By contrast, when available, participant-level characteristics, such as ethnicity, age, and BMI, explained a greater proportion of variance.

### 3.4 Vaginal microbiome species associated with health status

Using ANCOM-BC, we adjusted for available covariates such as age, BMI, and ethnicity to identify differential abundant taxonomies associated with disease status (**Figure 2C**). Across all datasets, various species of *Peptoniphilus* were consistently associated with disease status. Specifically, we observed enrichment of *Peptoniphilus urinimassilliensis* in the Walsh dataset*, Peptoniphilus coxii* in the Antonio and Chao datasets, and *Peptoniphilus* sp000478985 and sp900099555 in the Tsementzi and Gressel datasets, respectively. Notably, *Peptoniphilus coxii* and sp900099555 were also associated with BMI and age, respectively, in the Antonio and Walsh dataset. In addition to *Peptoniphilus*, species from the genera *Prevotella*, *Streptococcus*, and *Blautia* were differentially abundant across four of the five datasets, with the exception of Walsh et al., where these taxa were instead associated with BMI or ethnicity, both known EC risk factors.

Differentially abundant taxa reported in the original studies could not be reproduced using the published bioinformatics pipelines but were consistently recovered using the DADA2 pipeline. The DADA2 pipeline consistently outperformed other methods in identifying consistent ecological trends and was therefore selected as the primary preprocessing pipeline for model development.

### 3.5 Model development and validation

Four studies (Chao, Gressel, Tsementzi, and Walsh) were used for model training and one for performance evaluation (Antonio). Various data integration and modeling strategies were assessed, as outlined in **Figure 1**. Performance metrics for all ensemble models are detailed in **Table 2** and **Supplementary Section 9**.

**Table 2:** Performance metrics for early integration (non-batch corrected and ComBat batch-corrected) and late integration ensembles (microbiome data only, participant characteristics only, and participant characteristics and microbiome data) and associated 95% confidence intervals.

Model	NPV	Sensitivity	PPV	Specificity	AUROC
*Early integration:*					
*Non-batch corrected data*	NaN [0, 1]	1 [0.74, 1]	0.55 [0.32, 0.76]	0 [0, 0.31]	0.49 [0.23, 0.49]
*Batch-corrected data*	NaN [0, 1]	1 [0.74, 1]	0.55 [0.32, 0.76]	0 [0, 0.31]	0.5 [0.5, 0.5]
*Late integration ensemble:*					
*Microbiome data only*	0.64 [0.31, 0.89]	0.67 [0.35, 0.90]	0.73 [0.39, 0.94]	0.7 [0.35, 0.93]	0.63 [0.38, 0.63]
*Participant characteristics only*	0.69 [0.39, 0.91]	0.67 [0.35, 0.90]	**0.89 [0.52, 1]**	**0.9 [0.56, 1]**	0.88 [0.74, 0.88]
*Participant characteristics and vaginal pH*	0.64 [0.35, 0.87]	0.58 [0.28, 0.85]	0.88 [0.47, 1]	0.9 [0.56, 1]	0.87 [0.71, 0.87]
*Participant characteristics and microbiome data*	**1 [0.59, 1]**	**1 [0.74, 1]**	0.8 [0.52, 0.96]	0.7 [0.35, 0.93]	**0.93 [0.71, 0.93]**

NaN, Undefined, NPV, Negative Predictive Value, PPV, Positive Predictive Value, AUROC, Area Under Receiving Operating Characteristic curve (AUROC)

The best-performing model for each metric is indicated in bold.

Early integration models, which pooled data prior to modeling, showed limited ability to correctly identify benign cases. Batch correction using ComBat removed study-specific microbiome clustering (adonis PERMANOVA) while preserving variance due to disease status (see **Supplementary Section 10****).** However, models trained on batch-corrected or uncorrected data performed similarly in identifying EC cases, both achieving perfect sensitivity, but very poor specificity (0; 95% CI; [0, 0.31]), which resulted in undefined NPV.

Late integration models using only microbiome data achieved moderate performance in a held-out test set (sensitivity: 0.67 (95% CI; 0.35-0.90) and NPV: 0.64 (95% CI; 0.31-0.89)). Predicting with participant metadata where available: age, BMI, and ethnicity for Tsementzi and Walsh; age only for Chao—improved specificity 0.9 (95% CI; 0.56-1) but reduced sensitivity to 0.67 (95% CI; 0.35-0.90) and NPV to 0.69 (95% CI; 0.39-0.91). Including vaginal pH, a downstream biomarker of microbiome shifts, with other patient characteristics, did not enhance predictive value (**Table 2**).

The highest-performing model was an ensemble approach that integrated both microbial and host characteristics where available. Applied to the held-out test set, this model achieved perfect sensitivity of 1.0 (95% CI; 0.74-1) and NPV of 1.0 (95% CI; 0.59-1), with a specificity of 0.7 (95% CI; 0.35-0.93) and AUROC of 0.93 (95% CI; 0.71-0.93). Feature importance analysis across ensemble frameworks (**Supplementary Section 11**) identified *Lactobacillus*, *Prevotella*,

*Peptoniphilus*, *Porphyromonas*, *Peptostreptococcus*, and *Streptococcus* among the top 10 predictors, consistent with ANCOM-BC findings.

The LOSO validation of the ensemble model resulted in a pooled AUROC of 0.7 (95% CI; 0.6-0.7), ranging from 0.7 to 1.0 across individual studies, except for Tsementzi et al., which had the lowest data quality and an AUROC of 0.5 (**Supplementary Section 12**). The validation on the Chao et al. dataset, achieving perfect discrimination (AUROC of 1.0; 95% CI: [1-1]) and an NPV of 1.0 (95% CI: [0.9-1]), while the discrimation on the Walsh et al. and Gressel et al. datasets was moderate, with AUROC values of 0.8 (95% CI: [0.7-0.8]) and 0.7 (95% CI: 0.4-1), and NPVs of 0.8 (95% CI: 0.7-0.9) and 0.6 (95% CI: 0.4-0.8), respectively.

## 4 Discussion

In this study, we leveraged publicly available 16S rRNA gene sequencing data from five cohorts to evaluate the potential of vaginal microbiome data for a non-invasive screening approach for EC. We assessed the reproducibility and replicability of published findings and developed ensemble machine-learning models integrating microbial and host-specific data to predict EC status.

Our findings align with prior work emphasizing reproducibility challenges in microbiome research (Schloss, 2018; Kang et al., 2021; Rojas-Velazquez et al., 2024). These limitations often stem from a lack of standardized protocols, incomplete reporting of normalization techniques, and inconsistencies in the versions and parameter settings of the bioinformatics tools used. Additionally, participant-specific characteristics like age, BMI, and ethnicity, all known to strongly influence vaginal microbiome (Ravel et al., 2011; Hickey et al., 2012; Si et al., 2017; Łaniewski et al., 2020), are frequently unreported and unaccounted for in analyses. Reporting guidelines, such as the “Strengthening the Organization and Reporting of Microbiome Guidelines”, which outline reporting standards for microbiome studies, remain underutilized despite their potential to improve transparency and reproducibility (Mirzayi et al., 2021).

While we successfully recapitulated broad biological patterns, such as increased alpha diversity in EC cases, the magnitude and statistical significance of these trends varied across bioinformatics pipelines. Alpha diversity appeared particularly sensitive to preprocessing, reinforcing the importance of rigorous, consistent data filtering (Nearing et al., 2018; Kang et al., 2021). In contrast, beta diversity measures were more robust, revealing that host characteristics, rather than disease status, explained a greater proportion of variance across all datasets. These findings highlight the dominant influence of inter-individual variability on disease-driven microbiome shifts and underscore the need to incorporate participant metadata into analytical models.

We identified multiple species of *Peptoniphilus* as enriched in EC cases, consistent with prior findings from Walther-António et al., 2016; Tsementzi et al., 2020. Interestingly, *Peptoniphilus* was also associated with non-cancer traits, such as menopausal status and high vaginal pH, in the Walsh et al., 2019 data. These findings suggest that the observed associations with EC would be a consequence of local inflammation. This genus, a Gram-positive anaerobic coccus commonly found on mucosal surfaces, has previously been implicated in bacterial vaginosis and several gynecologic cancers (Diop et al., 2019; Wang et al., 2022; Asangba et al., 2023; Fong Amaris et al., 2024), as well as cancer of the mouth and gastrointestinal tracts (Murphy and Frick, 2013).

We additionally observed that various species of *Prevotella*, *Streptococcus*, and *Blautia* were associated with disease status across multiple datasets. Tsementzi et al. reported an association between *Prevotella* and cancer (Tsementzi et al., 2020). Species within this genus are commonly linked to HPV infections and are known to drive chronic mucosal inflammation, leading to tissue damage and potentially promoting oncogenesis (Larsen, 2017; Dong et al., 2022). Similarly, *Streptococcus* species can also act as pathogens by producing pro-inflammatory cytokines and activating carcinogenic pathways (Biarc et al., 2004; Abdulamir et al., 2009, Abdulamir et al., 2010; Kumar et al., 2017). Lastly, Antonio et al. found that *Blautia* was enriched in benign specimens and was associated with good outcomes, as evidenced by its inverse correlation with obesity and its ability to alleviate metabolic syndrome (Liu et al., 2021).

Final classifiers were validated and compared in an independent held-out dataset. We observed that batch correction techniques may overcorrect genuine biological signals (such as age, ethnicity, and varying inclusion criteria across studies), when these factors are not explicitly modeled. Early integration with batch correction yielded a sensitivity of 1 and a specificity of 0, underscoring the risk of overcorrection when relevant metadata are inconsistently available across cohorts.

In contrast, our late-integration ensemble model, which incorporates participant characteristics when available, achieved an AUROC of 0.93, NPV of 1, and a sensitivity of 1, demonstrating a strong potential utility for ruling out EC in symptomatic individuals. Although the small test set size (n = 22) yielded wide confidence intervals, these metrics indicate strong potential to rule out EC in individuals classified as negative without undergoing an endometrial biopsy. The model showed moderate specificity (0.7) and PPV (0.8), likely reflecting false positives among participants with undiagnosed gynecologic conditions. The overlap between classifier-derived feature importance and ANCOM-BC results strengthens the reliability of our findings. Using a LOSO validation, our framework yielded a pooled AUROC of 0.7, an NPV of 0.7, a sensitivity of 0.8, and a PPV of 0.6. Pooled LOSO validation was lower than when tested on Antonio et al., likely due to differences in cohort composition, missing demographic data, and primer variability.

Expanded training datasets that include participants with a broader range of benign conditions may improve specificity and PPV. Furthermore, transfer learning, a method that applies generalized patterns from diverse datasets to smaller, task- and cohort-specific tasks (Chong et al., 2022), could enhance classifier robustness. This approach could greatly benefit microbiome studies that often face data sparsity. For example, a colorectal cancer detection model trained on gut microbiome profiles from 20 different disease states outperformed one trained solely on colorectal cancer data, with an AUROC of 0.97 vs 0.6 (Chong et al., 2022).

Our study had several strengths. To our knowledge, this is the first EC prediction model using machine learning on 16S rRNA gene amplicon data across multiple cohorts (n = 243). All data were pre-processed using a uniform DADA2 pipeline, and individual-level patient characteristics were integrated where available. The classifier was validated using both a LOSO framework and in a single held-out test cohort, increasing confidence in its generalizability. However, limitations remain. The included cohorts primarily represent White participants, whereas EC disproportionately affects individuals from historically marginalized populations. For example, individuals who are Black, with obesity, single or widowed, have lower educational attainment, or live in rural areas, experience significantly higher incidence and mortality rates (Doll et al., 2020; Rodriguez et al., 2021). The lack of racial and socioeconomic diversity in microbiome studies risks perpetuating existing health disparities and should be addressed in future research. Additionally, differences in study protocols, such as the primers used, exclusion of participants on hormonal therapy in some studies (Tsementzi and Gressel) but not in others, may have introduced bias. Hormonal therapy is known to impact the composition of the vaginal microbiome, yet we were unable to adjust for this factor due to missing data.

If implemented as a screening tool, model performance should be evaluated in the same context as future applications —for example, in individuals experiencing AUB, the most common indication for an endometrial biopsy. Although specimens in our source studies were physician-collected, previous research suggests self-collected vaginal swabs are both sensitive and acceptable, offering a promising route for non-invasive screening (Costas et al., 2019; Camara et al., 2021).

Lastly, our predictive model was developed using 16S rRNA gene amplicon sequence data. Future work should investigate whether alternative sequencing approaches, such as metagenomics or targeting other amplicons, such as the chaperonin gene (cpn60), can improve model performance. For example, shotgun metagenomics has been shown not only to distinguish between benign and malignant conditions but also to predict EC grade and stage (Hakimjavadi et al., 2022).

In conclusion, accurately identifying individuals who require an endometrial biopsy remains a challenge. While several studies have reported vaginal microbial signatures of EC, this is the first to integrate microbial and host data across cohorts using a machine-learning framework. Our ensemble model reliably identified EC cases, demonstrating high sensitivity and negative predictive value. Our findings support integrating microbial features and host characteristics to enable robust prediction of EC status and underscore the potential of microbiome-based screening tools. These results could be achieved through non-invasive, self-collection methods that may broaden access to early detection and interventions. Future research should focus on validating models across diverse populations and real-world clinical settings.

## Data Availability

Publicly available datasets were analyzed in this study. This data can be found here: Sequence Read Archive with accession IDs: PRJNA295859, PRJNA481576, PRJNA448161, PRJNA758386, PRJNA843535.

## Footnotes

1
https://sourceforge.net/projects/bbmap/

2
https://github.com/TalhoukLab/VM01_EC_predictive_signature.git

## Supplementary material

Supplementary Section 1 Search keywords.

Supplementary Section 2 Data lost due to pipeline specifications.

Supplementary Section 3 Read length maintained after quality filtering.

Supplementary Section 4 Pipelines implemented.

Supplementary Section 5 TRIPOD + AI checklist

Supplementary Section 6 Grids used for hyperparameter optimization.

Supplementary Section 7 Detailed summaries of papers included.

Supplementary Section 8 Beta diversity estimates using various distance metrics.

Supplementary Section 9 Confusion matrices for models implemented.

Supplementary Section 10 Batch correction

Supplementary Section 11 Variable importance plots: Variable importance for microbiome models.

Supplementary Section 12 Leave-one-study-out (LOSO) validation results.
