# Supplementary material for: Multi-cohort ensemble learning framework for vaginal microbiome-based endometrial cancer detection
Source: Front Cell Infect Microbiol. 2025 Dec 8;15:1641413. doi: 10.3389/fcimb.2025.1641413 (PMC12719438; doi:10.3389/fcimb.2025.1641413)
Supplement: Supplementary Section 1 — Search keywords. [file DataSheet1.docx]

Supplementary Material

# Section 1: Search keywords

Search terms included: ((((((*Endometrial cancer*) OR (*Endometrial Neoplasms**)) OR (*Endometrial Hyperplasia**)) OR (*uterine cancer*)) OR (*uterine neoplasm**)) OR (*genital neoplasm**)) AND ((*vaginal microbiome*) OR (*uterine microbiome*)).

# Section 2: Data lost due to pipeline specifications

There was removal of samples due to either pipeline specific issues or lack of metadata available.

**Samples removed due to lack of metadata available**: 1 sample from Antonio (SRR2533882), 8 samples from Tsementzi (SRR6920030, SRR6920038, SRR6920042, SRR6920074, SRR6920057, SRR6920064, SRR6920070, SRR6920071), and 2 samples from the Walsh dataset (SRR7636336, SRR7636349).

**For the Antonio/Walsh pipeline**, we additionally lost 4 samples in the Antonio cohort as their (SRR2533873, SRR2533888, SRR2533965, SRR2533972) did not cluster with any OTUs.

**For the Chao pipeline**, Antonio lost additional 10 samples while quality filtering and Walsh lost an addition sample.

**For Gressel pipeline**, Antonio lost one additional sample (SRR2533933) during quality filtering.

**For Tsementzi pipeline**, Antonio lost 6 samples (SRR2533965, SRR2533972, SRR2533979, SRR2533985", SRR2533933, SRR2534037) during quality filtering, 4 (SRR2534046, SRR2534057, SRR2534013, SRR2533846) were lost due to no reads clustered in any OTUs. Tsementzi lost one additional sample during quality filtering (SRR6920035). Walsh lost 11 samples due to quality filtering (SRR7635466, SRR7635481, SRR7635605, SRR7635613, SRR7635688, SRR7635731, SRR7635879, SRR7635891, SRR7635971, SRR7636634, SRR7636737), additional 9 due to no clustering (SRR7635667, SRR7635862, SRR7635489, SRR7637034, SRR7635708, SRR7635964, SRR7635793, SRR7636849, SRR7635903).

**For DADA2 pipeline**,,Chao lost an additional sample (SRR19509417), Gressel lost 2 during DADA2 pre filter (SRR15655238, SRR15655294).

# Section 3: Read length maintained after quality filtering

Read length maintained for each study post trimming 5’ ends where the average phred quality score was below 20.

| **Study** | **Forward read length (base pairs)** | **Reverse read length (base pairs)** |
| --- | --- | --- |
| **Antonio** | 270 | 190 |
| **Chao** | 240 | 230 |
| **Gressel** | 250 | 200 |
| **Tsementzi** | 220 | 190 |
| **Walsh** | 220 | 190 |

**
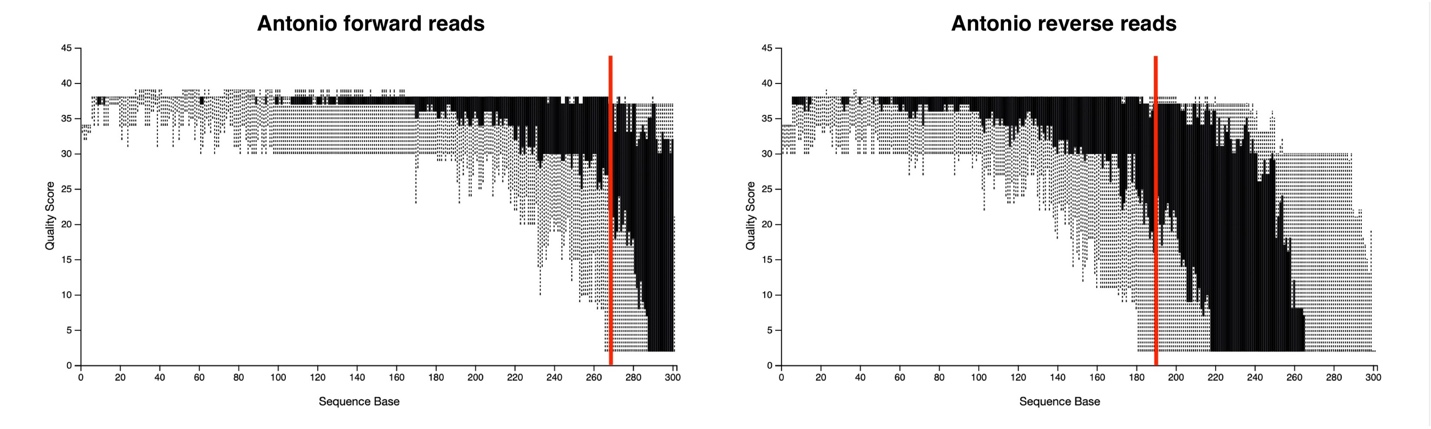
**

**
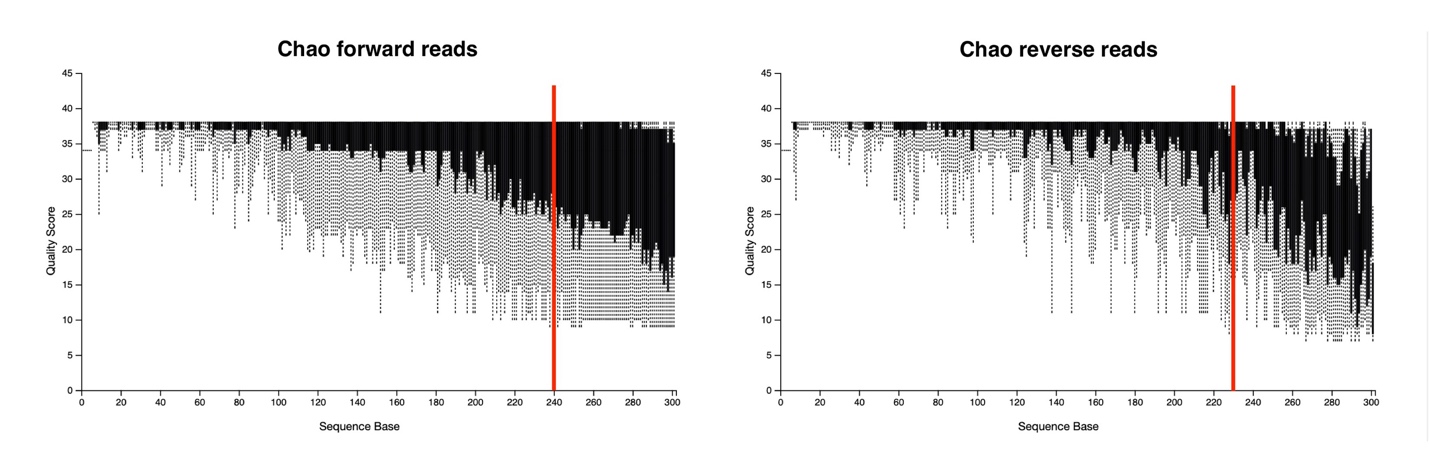
**

**
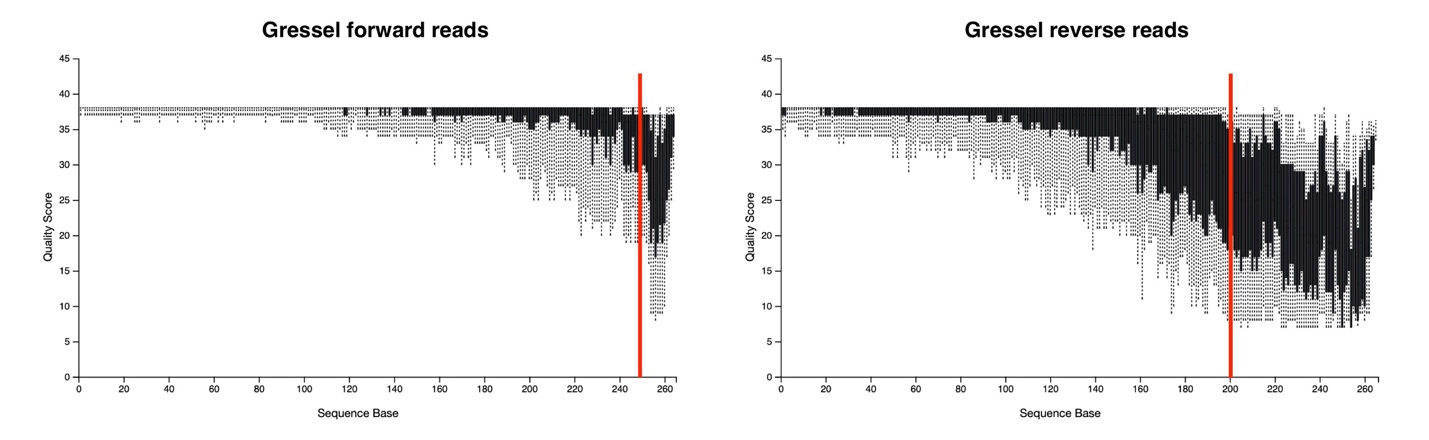
**

**
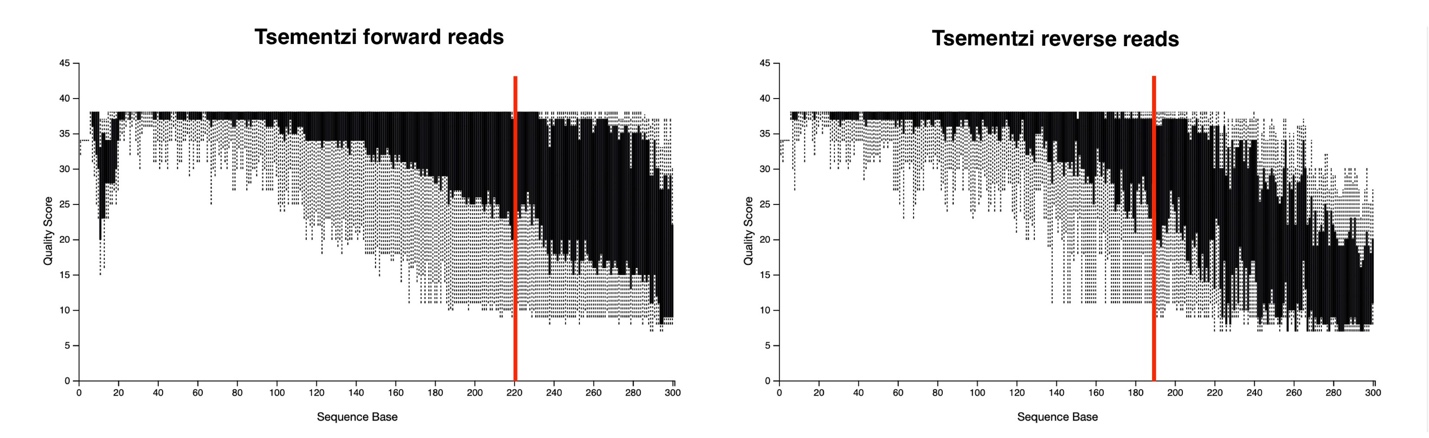
**

**
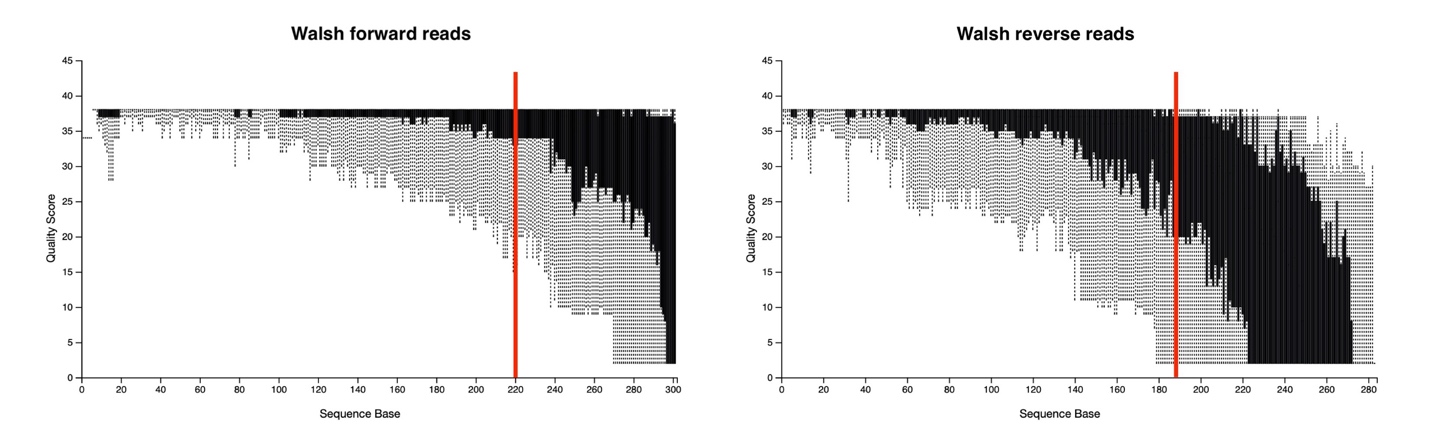
**

Quality plots for each dataset (Antonio, Chao, Gressel, Tsementzi, and Walsh) generated by QIIME2. The red lines indicate the read length maintained after trimming the 5’ ends of the reads when the average score is below 20. Gressel is our best quality dataset having high quality scores across the length of the reads.

Summary statistics of reads merged per cohort

|  | Antonio | Walsh | Tsementzi | Gressel | Chao |
| --- | --- | --- | --- | --- | --- |
| Mean % reads merged with > 12bp overlap | 0 | 5.71 | 3.42 | 80.6 | 34.6 |
| Proportion of samples with 50% of reads merged | 0 | 0 | 0 | 92.9 | 22.6 |

# Section 4: Pipelines implemented

All microbiome processing pipelines can be divided into five main steps: (1) preprocessing which includes (a) adapter/primer removal, (b) quality filtering, (c) paired-end merging, (d) dereplication of reads, and (e) chimera removal; (2) clustering reads into operational taxonomic units (OTUs) identified by a representative read (usually the centroid of the cluster) or denoising sequences to generate Amplicon Sequence Variants (ASVs); (3) taxonomic classification of representative reads or ASVs using classifiers and databases such as the Ribosomal Database project (RDP), Naïve Bayes (NB), SINTAX, GreenGenes (gg), and Genome Taxonomic Database. This table describes the pipelines implemented. If no parameters were listed by authors, we used default parameters. Specific versions of each tool used are highlighted.


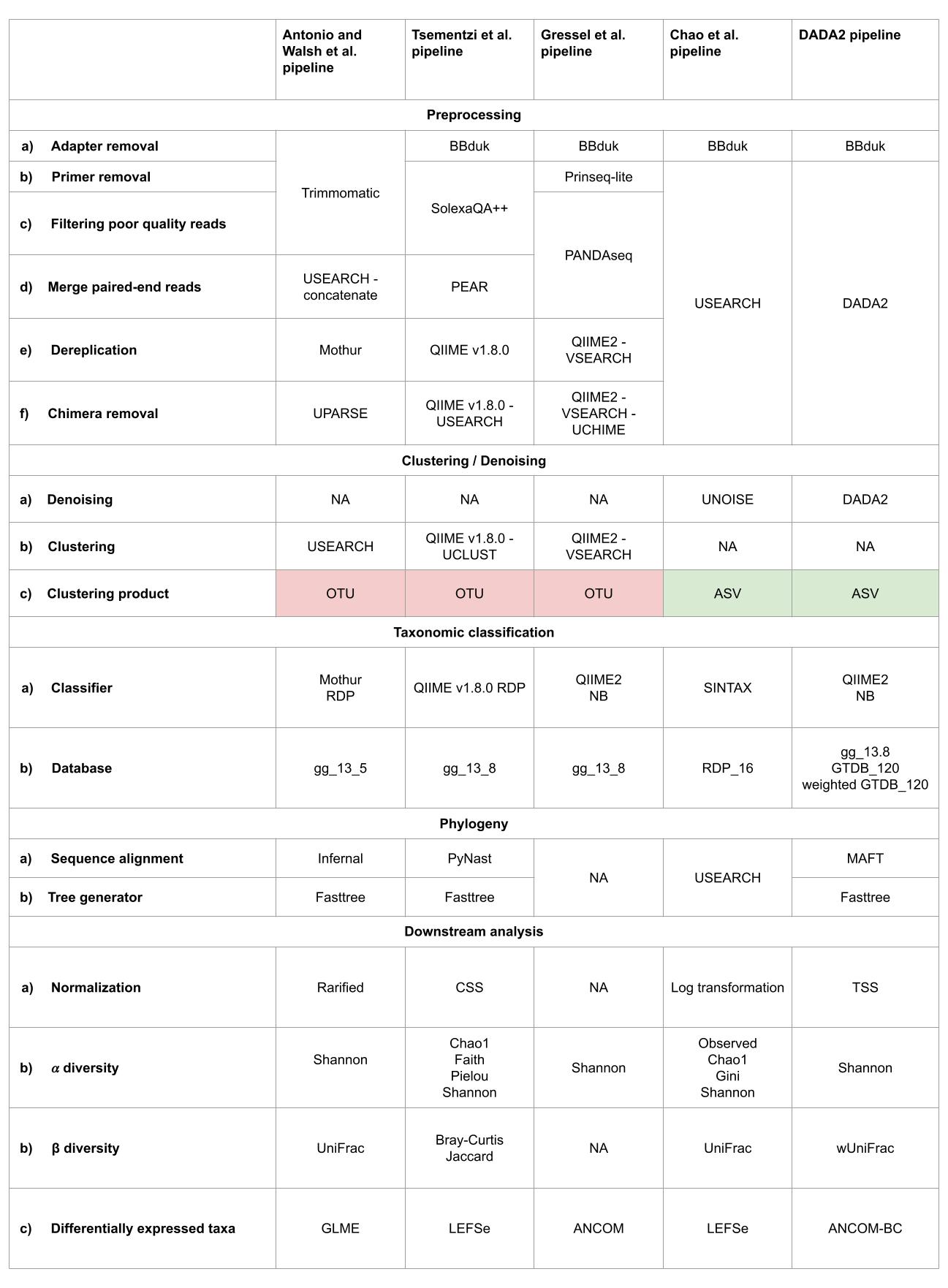


# Section 5: TRIPOD+AI checklist


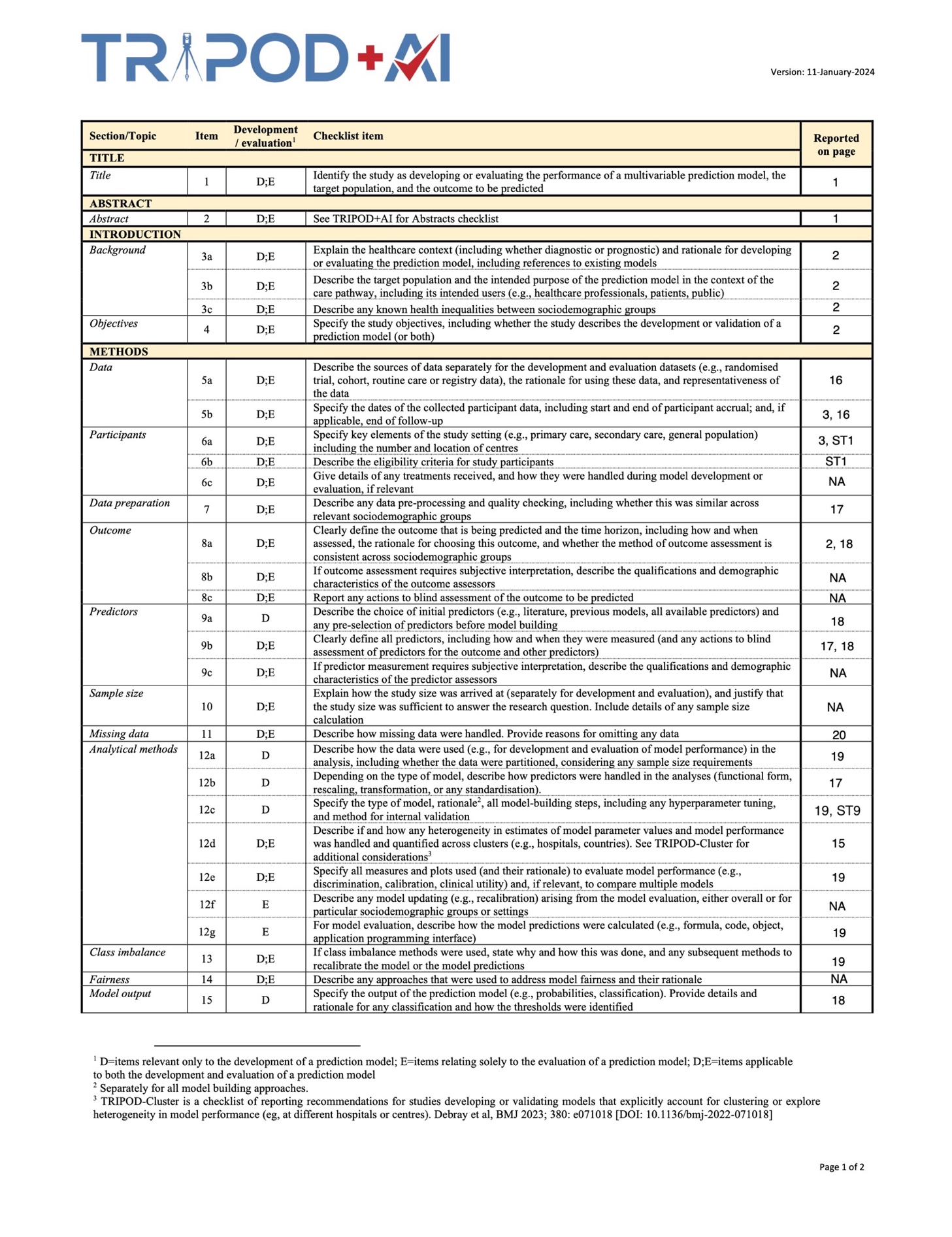


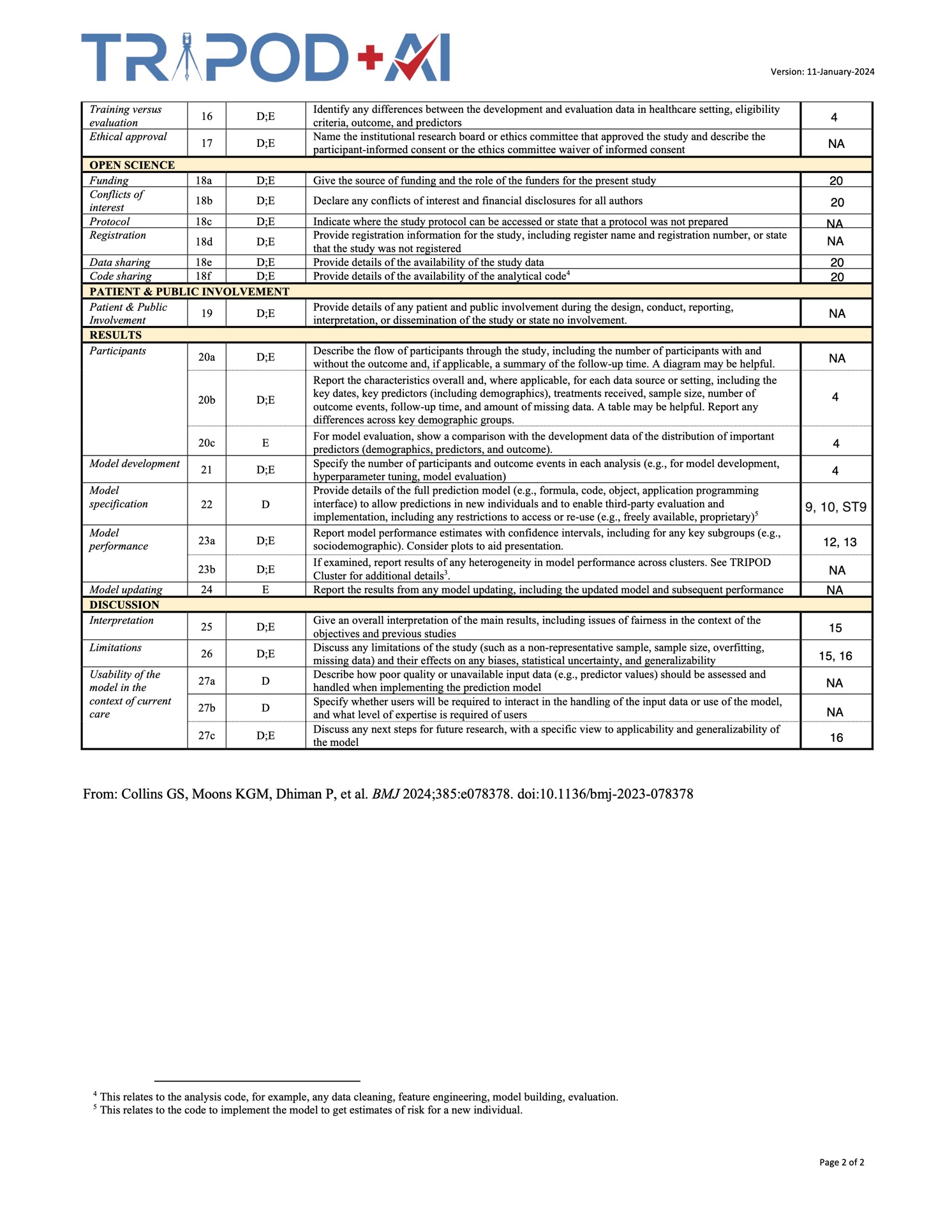


**Section 6: Grids used for hyperparameter optimization**

1. **Early integration – non-batch corrected data**
   1. **RF:** expand.grid(mtry = seq(150, 300, 10), min_n = seq(1, 15, 2), trees = seq(1500, 2500, 100))
   2. **NNET:** expand.grid(hidden_units = seq(10, 300, 10), penalty = seq(0, 1, 0.1), epochs = seq(5, 11, 2))
   3. **XGB:** expand.grid(mtry = seq(100, 300, 10), trees = 1500, min_n = seq(2, 10, 3), learn_rate = 0.3, loss_reduction = seq(0, 1, 0.5), stop_iter = Inf, tree_depth = seq(2, 15, 3), sample_size = c(0.5, 1))
2. **Early integration – ComBat batch-corrected data**
   1. **RF:** expand.grid(mtry = seq(150, 300, 5), min_n = seq(1, 15, 2), trees = seq(1500, 2500, 100))
   2. **NNET:** expand.grid(hidden_units = seq(10, 300, 10), penalty = seq(0, 1, 0.1), epochs = seq(5, 11, 2))
   3. **XGB:** expand.grid(mtry = seq(100, 300, 10), trees = 1500, min_n = seq(2, 10, 3), learn_rate = 0.3, loss_reduction = seq(0, 1, 0.5), stop_iter = Inf, tree_depth = seq(2, 15, 3), sample_size = c(0.5, 1))
3. **Late integration – microbiome only**
   1. **Walsh:**
      1. **RF**: expand.grid(mtry = seq(40, 150, 5), min_n = seq(1, 15, 2), trees = seq(1500, 2500, 100))
      2. **NNET:** expand.grid(hidden_units = seq(5, 150, 5), penalty = seq(0, 1, 0.1), epochs = seq(5, 11, 2))
      3. **XGB:** expand.grid(mtry = seq(1, 150, 10), trees = 1500, min_n = seq(2, 8, 2), tree_depth = seq(2, 15, 2), learn_rate = 0.002623724, loss_reduction = 9.899730e-04, sample_size = seq(0.5, 0.8, 0.1), stop_iter = 20)
   2. **Tsementzi:**
      1. **RF:** expand.grid(mtry = seq(40, 150, 5), min_n = seq(1, 15, 2), trees = seq(1500, 2500, 100))
      2. **NNET:** expand.grid(hidden_units = seq(5, 150, 5), penalty = seq(0, 1, 0.1), epochs = seq(5, 11, 2))
      3. **XGB:** expand.grid(mtry = seq(5, 150, 10), trees = 1500, min_n = seq(2, 10, 3), learn_rate = 0.3, loss_reduction = seq(0, 1, 0.5), stop_iter = Inf, tree_depth = seq(2, 15, 3), sample_size = c(0.5, 1))
   3. **Gressel:**
      1. **RF:** expand.grid(mtry = seq(30, 150, 5), min_n = seq(1, 15, 2), trees = seq(1500, 2500, 100))
      2. **NNET:** expand.grid(hidden_units = seq(5, 150, 5), penalty = seq(0, 1, 0.1), epochs = seq(5, 11, 2))
      3. **XGB:** expand.grid(mtry = seq(5, 150, 10), trees = 1500, min_n = seq(2, 10, 3), learn_rate = 0.3, loss_reduction = seq(0, 1, 0.5), stop_iter = Inf, tree_depth = seq(2, 15, 3), sample_size = c(0.5, 1))
   4. **Chao:**
      1. **RF:** rf_grid <- expand.grid(mtry = seq(15, 150, 10), min_n = seq(1, 15, 2), trees = seq(1500, 2500, 100))
      2. **NNET**: expand.grid(hidden_units = seq(5, 100, 5), penalty = seq(0, 1, 0.1), epochs = seq(5, 11, 2))
      3. **XGB:** expand.grid(mtry = seq(5, 150, 10), trees = 1500, min_n = seq(2, 10, 3), learn_rate = 0.3, loss_reduction = seq(0, 1, 0.5), stop_iter = Inf, tree_depth = seq(2, 15, 3), sample_size = c(0.5, 1))
4. **Early integration: participant characteristics only (with and without vaginal pH)**
   1. **Walsh:**
      1. **RF:** expand.grid(mtry = seq(1, 4, 1), min_n = seq(2, 10, 1), trees = seq(1500, 2500, 100))
      2. **NNET:** expand.grid(hidden_units = seq(1, 30, 1), penalty = seq(0, 1, 0.1), epochs = seq(5, 11, 2))
      3. **XGB:** expand.grid(mtry = seq(1, 4, 1), trees = 1500, min_n = seq(2, 10, 1), learn_rate = 0.3, loss_reduction = seq(0, 1, 0.5), stop_iter = Inf, tree_depth = seq(2, 15, 3), sample_size = c(0.5, 1))
   2. **Tsemenzti:**
      1. **RF**: expand.grid(mtry = seq(1, 4, 1), min_n = seq(2, 10, 1), trees = seq(1500, 2500, 100))
      2. **NNET:** expand.grid(hidden_units = seq(1, 30, 5), penalty = seq(0, 1, 0.1), epochs = seq(5, 11, 2))
      3. **XGB:** expand.grid(mtry = seq(1, 4, 1), trees = 1500, min_n = seq(2, 10, 1), learn_rate = 0.3, loss_reduction = seq(0, 1, 0.5), stop_iter = Inf, tree_depth = seq(2, 15, 3), sample_size = c(0.5, 1))
   3. **Chao:**
      1. **RF:** expand.grid(mtry = 1, min_n = seq(2, 10, 1), trees = seq(1500, 2500, 100))
      2. **NNET:** expand.grid(hidden_units = seq(5, 100, 5), penalty = seq(0, 1, 0.1), epochs = seq(5, 11, 2))
      3. **XGB:** expand.grid(mtry = 1, trees = 1500, min_n = seq(2, 10, 1), learn_rate = 0.3, loss_reduction = seq(0, 1, 0.5), stop_iter = Inf, tree_depth = seq(2, 15, 3), sample_size = c(0.5, 1))
5. **Late integration: participant characteristics and microbiome data (with and without vaginal pH)**
   1. **Walsh**
      1. **RF**: expand.grid(mtry = seq(5, 200, 1), min_n = seq(2, 12, 2), trees = 2000)
      2. **NNET:** expand.grid(hidden_units = seq(5, 150, 3), penalty = seq(0, 1, 0.1), epochs = seq(5, 11, 2))
      3. **XGB:** expand.grid(mtry = seq(5, 150, 3), trees = 1500, min_n = seq(2, 10, 3), learn_rate = 0.3, loss_reduction = seq(0, 1, 0.5), stop_iter = Inf, tree_depth = seq(2, 15, 3), sample_size = c(0.5, 1))
   2. **Tsementzi**
      1. **RF:** expand.grid(mtry = seq(5, 200, 1), min_n = seq(2, 12, 1), trees = 2000)
      2. **NNET:** expand.grid(hidden_units = seq(5, 100, 5), penalty = seq(0, 1, 0.1), epochs = seq(5, 11, 2))
      3. **XGB:** expand.grid(mtry = seq(5, 150, 10), trees = 1500, min_n = seq(2, 10, 3), learn_rate = 0.3, loss_reduction = seq(0, 1, 0.5), stop_iter = Inf, tree_depth = seq(2, 15, 3), sample_size = c(0.5, 1))
   3. **Chao**
      1. **RF:** expand.grid(mtry = seq(5, 150, 1), min_n = seq(2, 12, 2), trees = 2000)
      2. **NNET:** expand.grid(hidden_units = seq(5, 150, 3), penalty = seq(0, 1, 0.1), epochs = seq(5, 11, 2))
      3. **XGB:** expand.grid(mtry = seq(5, 100, 3), trees = 1500, min_n = seq(2, 10, 3), learn_rate = 0.3, loss_reduction = seq(0, 1, 0.5), stop_iter = Inf, tree_depth = seq(2, 15, 3), sample_size = c(0.5,1)

| **Section 7: Detailed summaries of papers included** | | | | | |
| --- | --- | --- | --- | --- | --- |
|  | **Antonio et al.** | **Walsh et al.** | **Tsementzi et al.** | **Gressel et al.** | **Chao et al.** |
| **Date of publication** | 2016 | 2019 | 2020 | 2021 | 2022 |
| **Journal** | Genome medicine | Scientific reports | Cancer Medicine | PLoS One | Frontiers in cellular and infection microbiology |
| **Inclusion criteria** | - 18 years of age or older. - Women undergoing hysterectomy by any standard surgical approach. - Undergoing hysterectomy for benign disease, hyperplasia, or any stage of endometrial cancer. | Same as antonio et al. | - Post-menopausal - Previous hpv or other std is not exclusionary if treated and cleared - **Well-controlled** type 2 diabetes is acceptable - Hypothyroidism **is not** exclusionary if controlled (i.e. Treated with medication) - Must be able to read, write, and speak english - Diagnosed with either **cervical** or **endometrial** cancer [for cancer cohort] - Scheduled for radiation therapy (prior surgery and/or chemotherapy is permitted) [for cancer cohort] | - English or spanish-speaking - Post-menopausal (defined as 50 years of age or older who have not had menses for 12 consecutive months or more) - Biopsy proven well-differentiated endometrioid endometrial adenocarcinoma (eac), uterine serous carcinoma (usc) - Or with non-cancerous conditions requiring hysterectomy. | Women with recent abnormal uterine bleeding due to a suspected endometrial lesion (e.g., polyp, myoma, EH, or EC) who were referred for an office hysteroscopy were eligible, as were those with either a newly diagnosed or known EH/early EC who had undergone fertility-preserving treatment |
| **Exclusion criteria** | - Women who were pregnant or nursing. - Had taken antibiotics within two weeks preceding surgery - Surgeon using morcellation during the hysterectomy procedure, due to the size of the uterus or for any other reason. | Same as antonio et al. | - History of metastatic or other primary cancer or previous radiation therapy - Co-morbidities including: hiv, cystic fibrosis, poorly controlled diabetes, autoimmune disease, stds, fungal infection, crohn’s disease, ulcerative colitis, hepatitis c, hsv (herpes simplex) - Exclude diabetic patients with neuropathy, history of gout, high bmi (>49), and/or taking neurontin (gabapentin)—**poorly controlled** - Type 1 diabetes - Use of hrt (hormone replacement therapy) and/or vaginal estrogens within 4 weeks of baseline - Use of antibiotics, corticosteroids, or topical estrogen within 4 weeks requires rescheduling or exclusion - Use of interferon(s) ***or*** immunosuppressive therapies or *megestrol* ex. Interferon beta-1a (i.e. Rebif, plegridy, etc.) | - History of prior cancer - prior chemotherapy or radiation therapy - Prior bariatric surgery - History of human immunodeficiency virus or pid - Use of douching, hormone therapy, systemic or local antibiotics, pro-biotics or anti-fungal medications within 2 weeks of initial consultation - Or confirmed urinary tract or vaginal infection such as bacterial vaginosis, sexually transmitted disease or candidiasis within 1 month of initial consultation. | Women with suspected endometritis and those unwilling to participate were excluded. |
| **Sample collection** | All participants were requested not to douche with betadine on the day of surgery or the day immediately preceding it. Vaginal swabs were collected by surgeons (with guidance from the research team) immediately after the administration of anesthesia and immediately preceding the standard pre-surgical betadine douche. Collected using Darcon swabs. | Same as Antonio et al. | Collected by physicians following the protocol by Human Microbiome Project. Briefly, used sterile catch all swabs (Epicentre Biotechnologies, Madison WI) to gently rub the mid-vaginal wall. | After induction of anesthesia and prior to antibiotic administration and vaginal preparation, the patient was placed in dorsal lithotomy position. A sterile bi-valve speculum was inserted inside the vagina to expose the uterine cervix and vaginal canal. A sterile q-tip applicator was used to swab the vaginal fornices | Prior to endometrial biopsies, normal saline was instilled to provide distension and irrigation of the uterine cavity. Endometrial lavage samples (25 mL) obtained using a continuous-flow rigid hysteroscopy system (sheath diameter: 4 mm; Richard Wolf GmbH, Knittlingen, Germany) |
| **Sample storage** | After collection, swabs were placed in sterile tube with 1 mL of Tris-EDTA (TE) buffer kept on dry ice until storage at –80 °C. | Same as Antonio et al. | Samples were obtained from the midvagina and stored in sterile MoBio Power Bead tubes (Mo Bio) at −80°C. | After collection, swabs were placed into a 1 mL tube of Specimen Transport Medium (STM) (Digene Female Swab Specimen Collection Kit, Qiagen, CA).  All research samples were stored in a -20° freezer within one hour of collection. | Immediately placed into sterile tube and centrifuged at 3200 rpm for 20 min at 4°C. Cell pellets were suspended in PBS, washed with a red blood cell lysis solution, incubated at room temperature for 30 min, and centrifuged at 3000 rpm for 10 min. After removal of the supernatant, pellets were stored -80°C until analysis |
| **Vaginal pH collection** | One extra swab was used for vaginal pH collection immediately after collection. Vaginal pH was measured using Hydrion measuring pH tape. | Same as Antonio et al. | Take at least two separate recordings (digital recordings of pH), and determine a mean pH. If the mean pH is ≤ 4.5, then eligibility is confirmed with regard to vaginal pH. | Not applicable | Not applicable |
| **Sample processing** | The swab samples were centrifuged for 10 min at 10,000 g to collect the bacterial cells and the supernatant was discarded. They were then centrifuged for 10 min at 10,000 g to collect the bacterial cells and the supernatant was discarded. | Same as Antonio et al. | Not specified | Modified by pre-incubation with Proteinase K and agitation with glass beads. Sterility was maintained by processing these samples in a sterile Biosafety Cabinet in an isolated extraction room. | See sample storage |
| **DNA extraction** | MoBio PowerSoil Kit (MoBio Laboratories, Inc., Carlsbad, CA, USA) as described by the manufacturer; however, instead of vortexing, an MP FastPrep (MP Biomedicals, Solon, OH, USA) was used instead, for 60 s at 6.0 m/s, to obtain a more effective and rapid lysis of the cells. | MoBio PowerSoil® DNA Isolation Kit (PN 12888 Mo Bio Laboratories, Inc. Carlsbad, CA) according to the manufacturer’s protocol. | DNeasy PowerSoil Kit (Qiagen). | QIAamp DNA Kit (Qiagen, CA) | QiaAmp DNA Microbiome Kit (Qiagen, Hilden, Germany). |
| **DNA content measurement** | High Sensitivity Qubit (Life Technologies Corporation, Carlsbad, CA, USA) | Qubit dsDNA HS Assay Kit (PN Q32854 Thermo Fisher Scientific Inc., Waltham, MA) | Not specified | Not specified | A Qubit dsDNA High Sensitivity Assay (Thermo Fisher Scientific, Waltham, MA, USA) was used to determine the concentration and quality of purified DNA. |
| **16S rRNA region** | V3-V5 | V3-V5 | V4 | V4 | V3-V4 |
| **Primer sequence provided** | F: 5’AATGATACGGCGACCACCGAGATCTACACTATGGTAATTGTCCTACGGGAGGCAGCAG3’  R: 5’CAAGCAGAAGACGGCATACGAGATGCCGCATTCGATXXXXXXXXXXXXCCGTCAATTCMTTTRAGT3’ | F:  TCGTCGGCAGCGTCAGATGTGTATAAGAGACAGCCTACGGGAGGCAGCAG  R:  GTCTCGTGGGCTCGGAGATGTGTATAAGAGACAGCCGTCAATTCMTTTRAGT | Not specified | Not specifed | F:  5’-TCGTCGGCAGCGTCAGATGTGTATAAGAGACAGCCTACGGGNGGCWGCAG-3’  R:  5’-GTCTCGTGGGCTCGGAGATGTGTATAAGAGACAGGACTACHVGGGTATCTAATCC-3’. |
| **Amplification** | Polymerase chain reaction as follows: 25 μL of Kapa HiFi (Kapa Biosystems, Woburn, MA, USA), 1.5 μL (10 uM) forward primer, 1.5 μL (10 uM) reverse primer, 50 ng of DNA with the remaining volume being added by molecular grade water (up to a final volume of 50 μL per reaction). | Two step PCR:   - First qPCT: Sample were amplified with the following conditions: 95 °C for 5 minutes, 25 cycles of: 98 °C for 20 seconds, 55 °C for 19 seconds, and 72 °C for 60 seconds, and a final 72 °C extension for 5 minutes. - Primary PCR products were diluted 1:100 in PCR grade water for secondary PCR reactions. | PCR amplification | PCR amplification | PCR amplification |
| **Contamination checks** | The products of the amplification were verified by a TapeStation D1K Tape (2200 TapeStation Instrument, Agilent Technologies, Santa Clara, CA, USA) to be free of contamination and to contain the expected amplification size, approximately 700 base pairs. If the amplification was unsuccessful, the parameters of the reaction or cycle were adjusted in repeated attempts. In samples that failed 16S rDNA amplification, NEBNext Microbiome DNA Enrichment Kit (New England Biolabs Inc., Ipswitch, MA, USA) was used to separate the microbiome from the human DNA to increase the odds of a successful amplification from samples naturally enriched with human DNA (mostly tissue samples). | PCR products were diluted to 20 uL with PCR grade water and cleaned up using 1.0X AMPureAP beads (Beckman Coulter, Brea, CA), vacuum-dried, reconstituted in 12 uL of PCR grade water, quantified using a Quant-It dsDNA HS assay kit (Thermo Fisher Scientific Inc., Waltham, MA), normalized and pooled. The sequencing pool was concentrated, cleaned up using 1.8X AMPureAP beads (Beckman Coulter, Brea, CA) quantified using a Quant-It dsDNA HS assay kit (Thermo Fisher Scientific Inc., Waltham, MA). Sequence pool was assessed for purity and the presence of 725 bp peak (±20%) using a 2200 TapeStation system and D1000 Screen tape/reagents (Agilent Technologies, Santa Clara, CA). | Not specified | Not specified | Not specified |
| **Sequencing platform** | Illumina MiSeq | Illumina MiSeq | Illumina MiSeq | Illumina MiSeq | Illumina MiSeq |
| **Bioinformatics pipeline** | IM-TORNADO | IM-TORNADO | UCLUST in QIIME v.1.8.0 | VSEARCH in QIIME2 | USEARCH |
| **Participant characteristics collected** | Age, ethnicity, BMI, menopausal status, gravida, parity, hypertension, diabetes, smoking status, vgainal pH, histotype, grade and stage | Same as Antonio et al. | Age, ancestry, diagnosis, and type of treatment | Age, BMI, parity, race, ethnicity, diabetes, hypertension, smoking status, and cancer stage | Health condition, age |
| **Participant characteristics available** | Same as above | Same as Antonio | Same as above | Health condition | Same as above |
| **Main results** | - **Alpha diversity:** significantly greater microbial diversity in EC vs benign (p-value 0.003) - **Beta diversity:** significant difference between EC and benign (p=0.01) - **EC associated taxa:** Atopobium vaginae and Porphyromonas sp. With high vaginal pH (> 4.5) | - **Alpha diversity:** greater microbial diversity in EC vs benign (p-value 0.417) - **Beta diversity:** significant difference between EC and benign (p=0.04) - **EC associated taxa:** Porphyromas somerae | - **Alpha diversity:** significantly greater microbial diversity in pre-RT cancer patients vs benign (q-value = 0.03) - **Beta diversity:** health condition (health vs cancer) explains 1% of observed variation in microbiome composition - **EC associated taxa:** Sneathia, Prevotella, Peptoniphilus, Fusobacterium, Anaerococcus, Dialister, Moryella, and Peptostreptococcus | - **Alpha diversity:** greater microbial diversity in EC vs benign (q=0.252) - **Beta diversity:** did not analyze by health condition - **EC associated taxa:** Pseudomonas | - **Alpha diversity:** significantly greater microbial diversity in EC vs benign (p-value – 0.03) - **Beta diversity:** significant differences between EC and benign (p-value < 0.05) - **EC associated taxa:** Bacillus pseudofirmus and Stenotrophomons rhizophila |

**Section 8: Beta diversity estimates using various distance metrics**


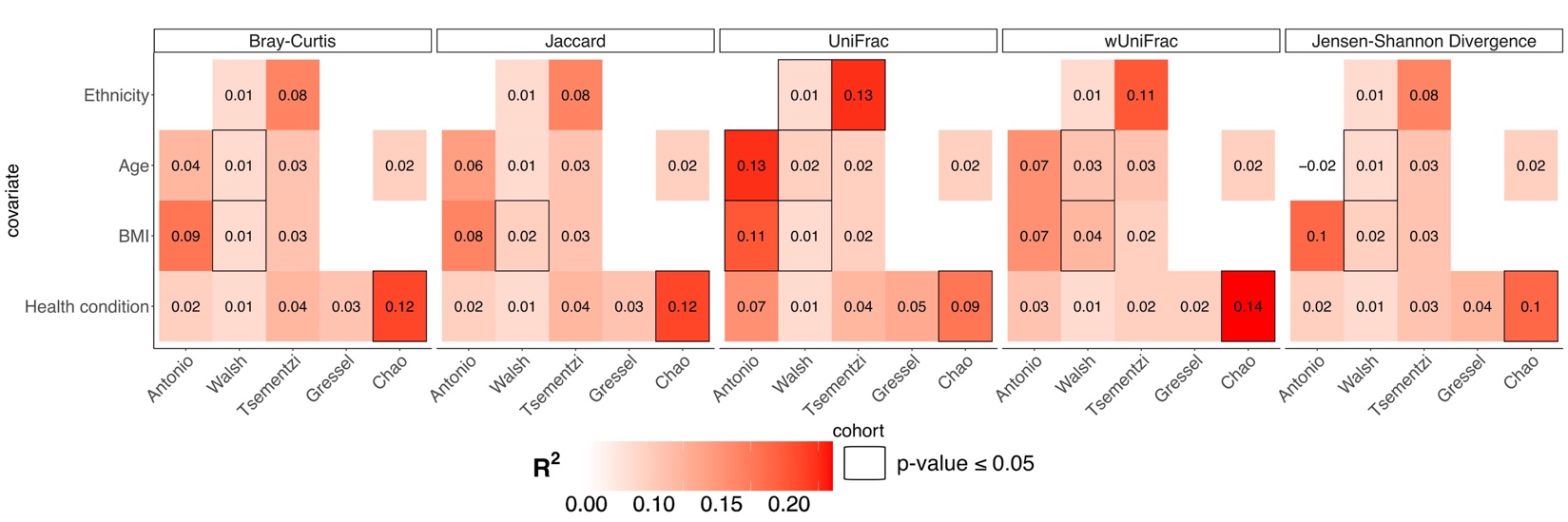


Each panel represents the marginal proportion of variance explained by participant characteristics (y-axis) available in each dataset when (x-axis) processed by the pipeline in the respective panel. Consistent trends were observed across pipelines. Health conditions explain less than 14% of variance in all datasets, whereas individual characteristics appear to have more influence on the structure and composition of the vaginal microbiome.

**Section 9: Confusion matrices for models implemented**


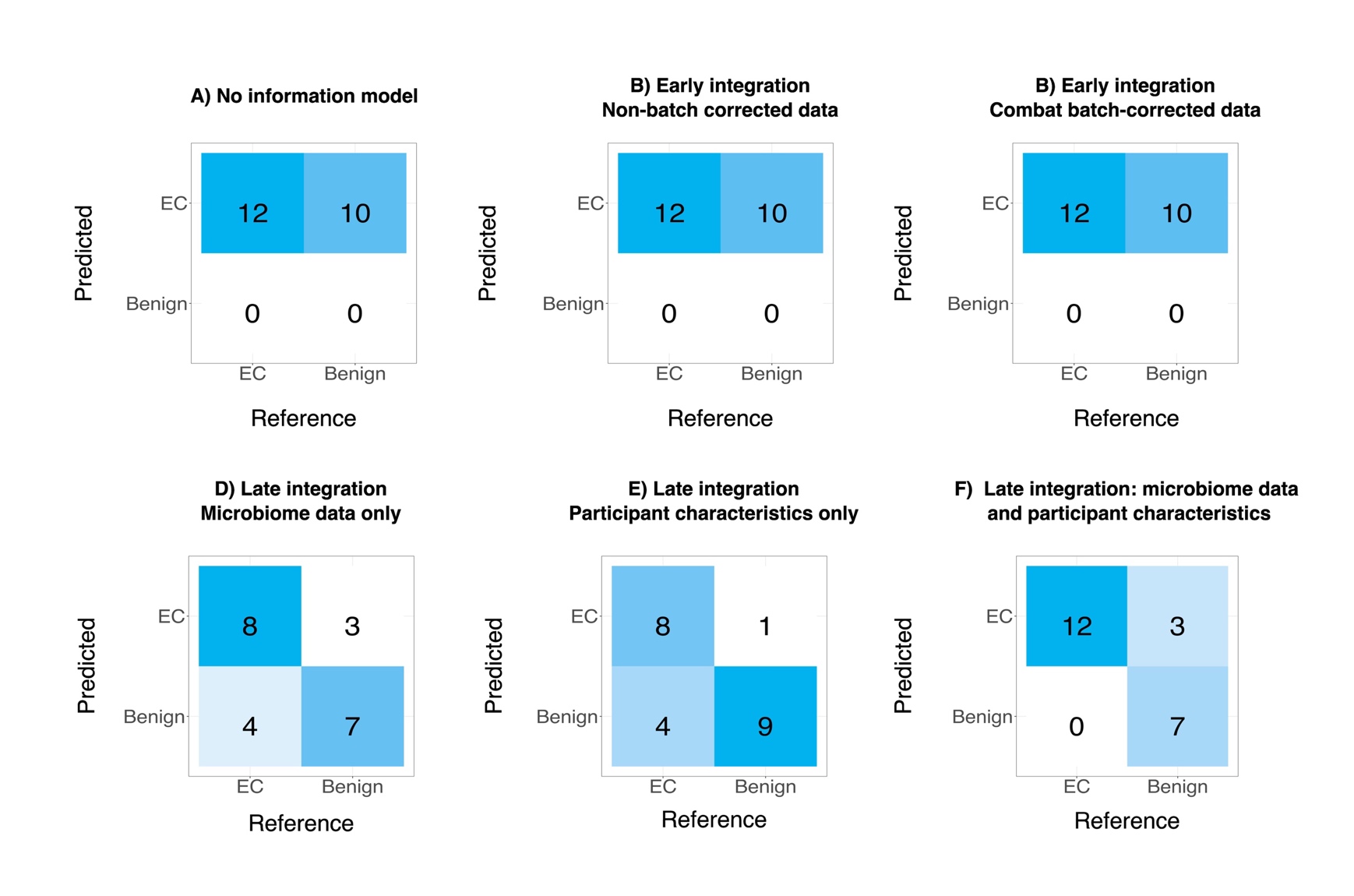


Confusion matrices for A) no information model where all test instances are assigned to the majority class (EC), early integration frameworks using B) non-batch corrected and C) ComBat batch-corrected data, late integration frameworks using D) microbiome data only, E) participant characteristics only, and F) microbiome data and participant characteristics.

**Section 10: Batch correction**


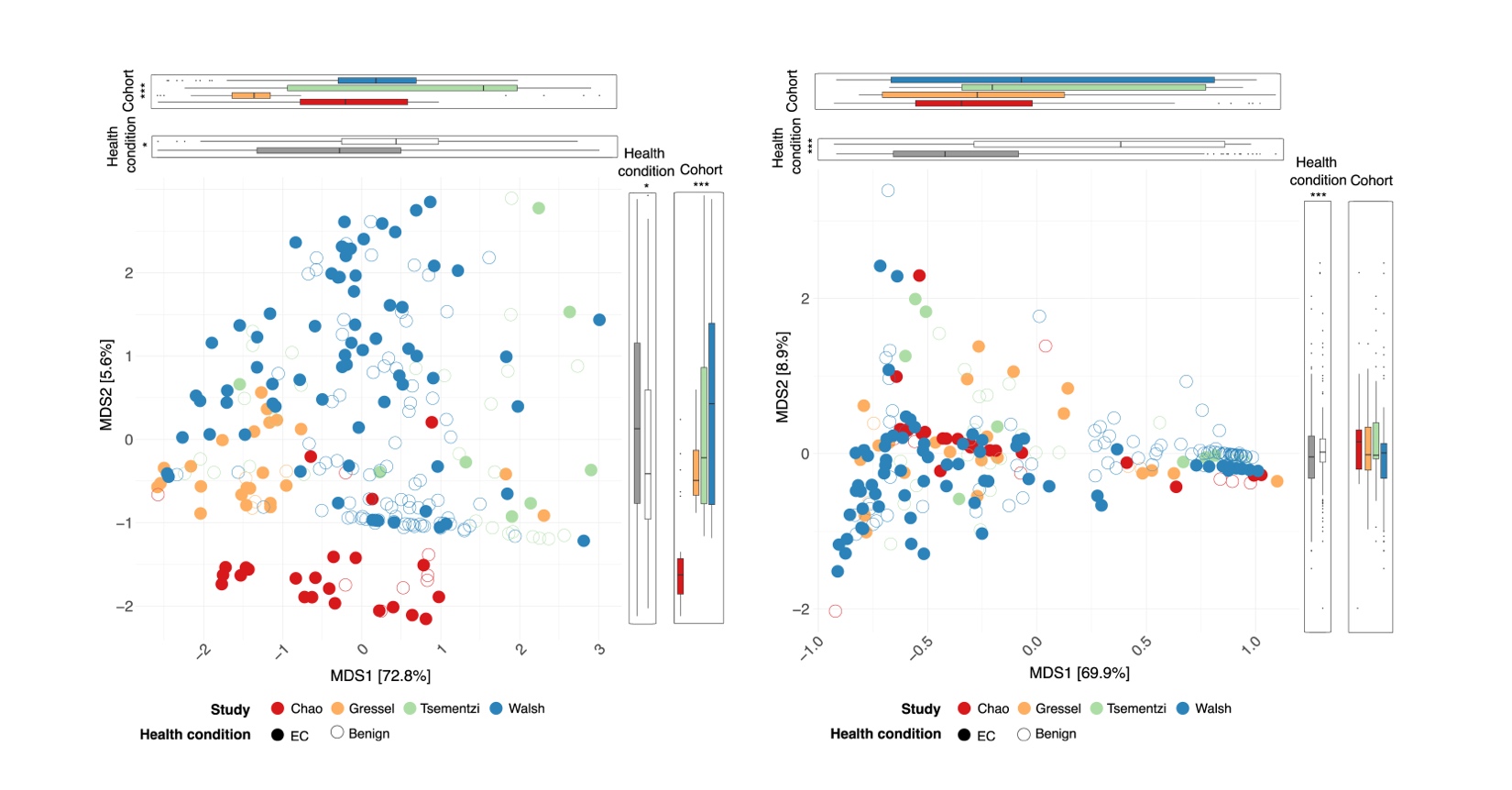


Adnois2 results before batch correction

|  | R2 | F | Pr(>F) |
| --- | --- | --- | --- |
| Cohort | 0.13430 | 13.0415 | 0.001*** |
| Histology | 0.01419 | 4.1329 | 0.023* |
| Residual | 0.81698 |  |  |
| Total | 1 |  |  |

Adnois2 results after batch correction

|  | R2 | F | Pr(>F) |
| --- | --- | --- | --- |
| Cohort | 0.01203 | 1.0828 | 0.333 |
| Histology | 0.09466 | 25.5639 | 0.001*** |
| Residual | 0.88132 |  |  |
| Total | 1 |  |  |

Comparison of batch effect correction in early integration frameworks. Distribution of participants in each study following principal coordinates analysis based on the relative abundance (at the genus level) of EC (filled points) and benign (open points) A) without batch effect correction and B) ComBat batch corrected. Box plots represent the distribution between studies or between health condition. There is significant difference between studies before batch correction (p-value < 0.001 using adnois2 PERMANOVA); this difference disappears after batch correction.

**Section 11: Variable importance plots :** Variable importance for microbiome models


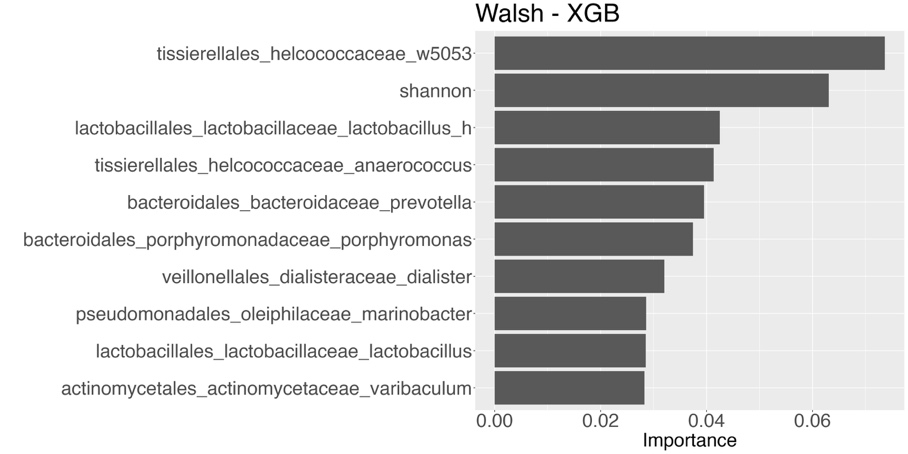

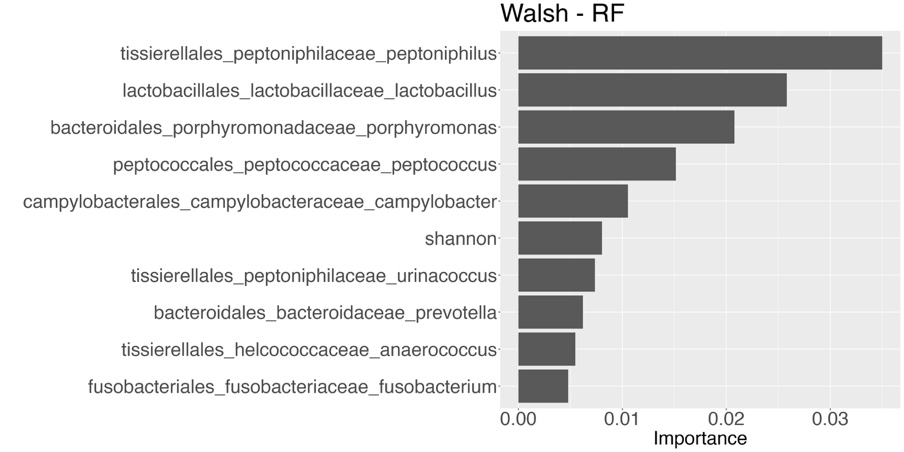

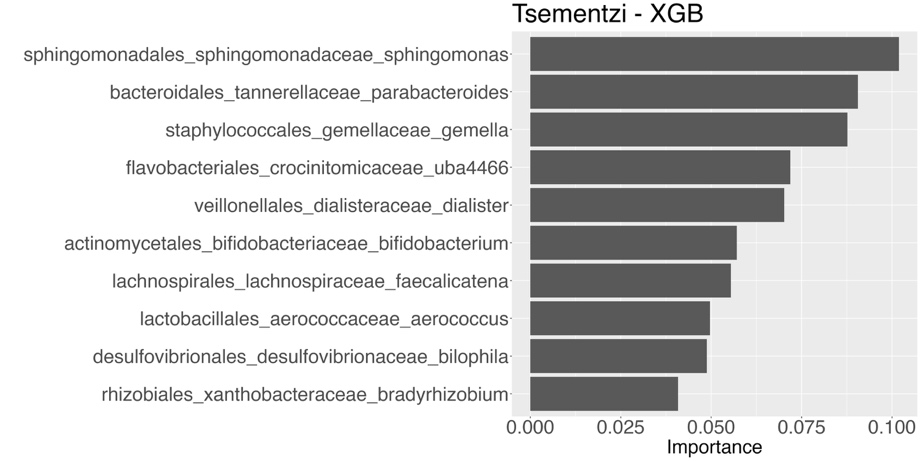

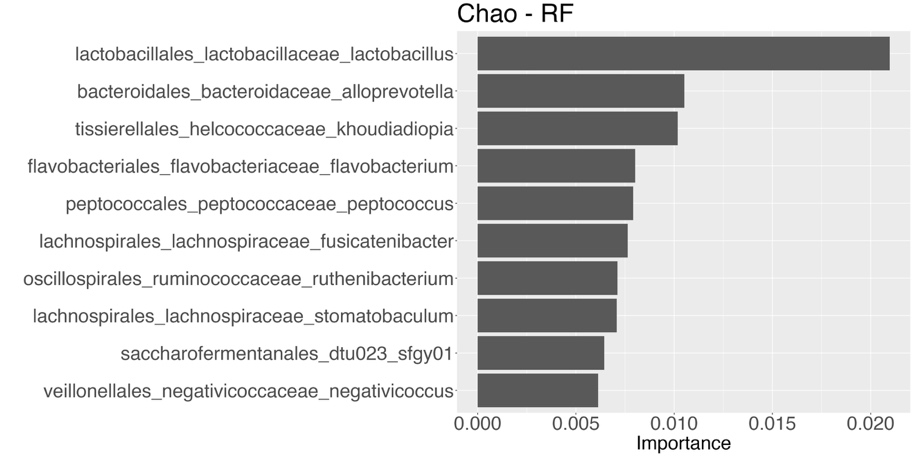

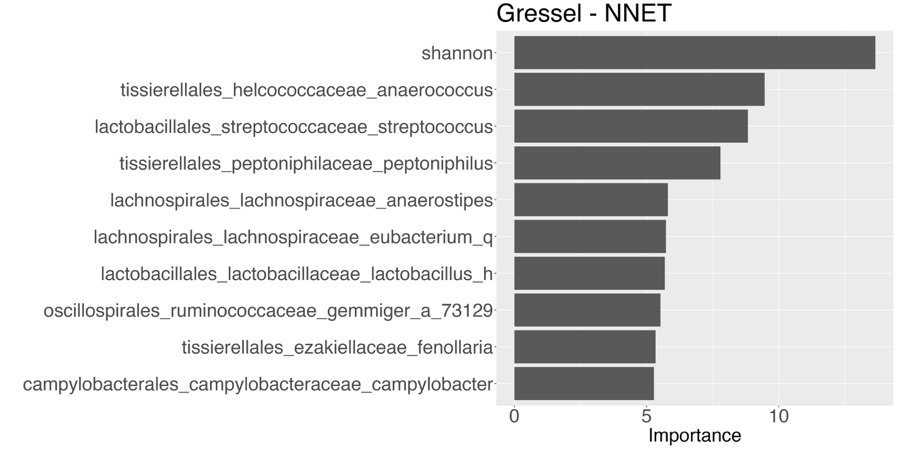


Variable importance for participant characteristics models (with pH)


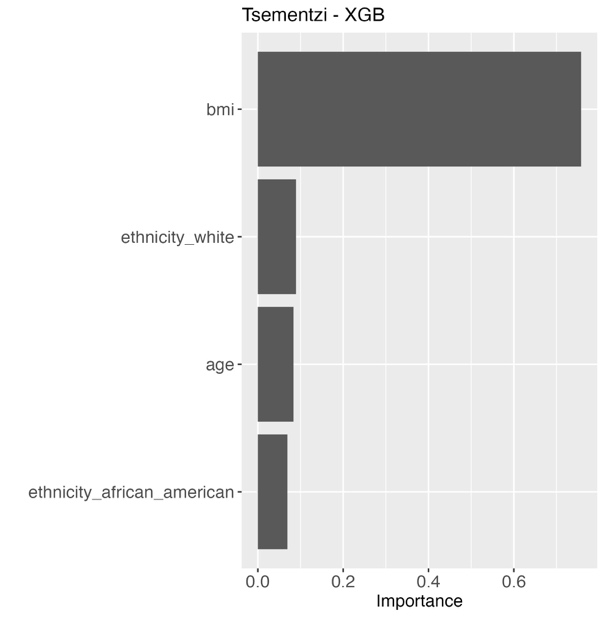

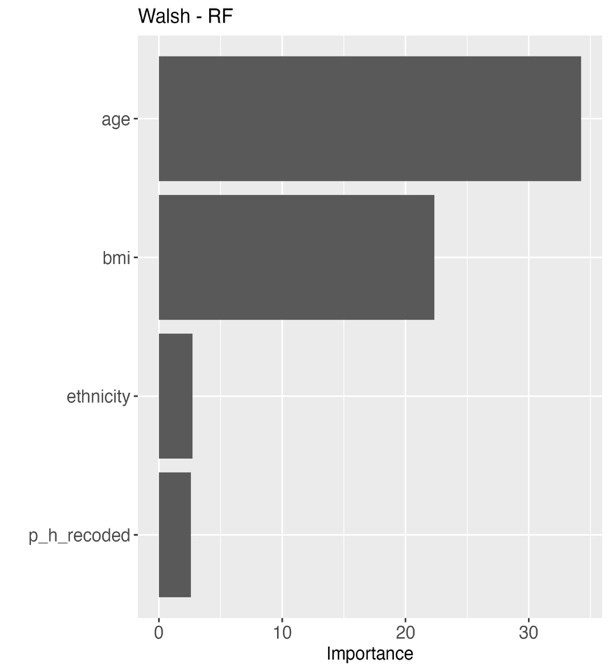

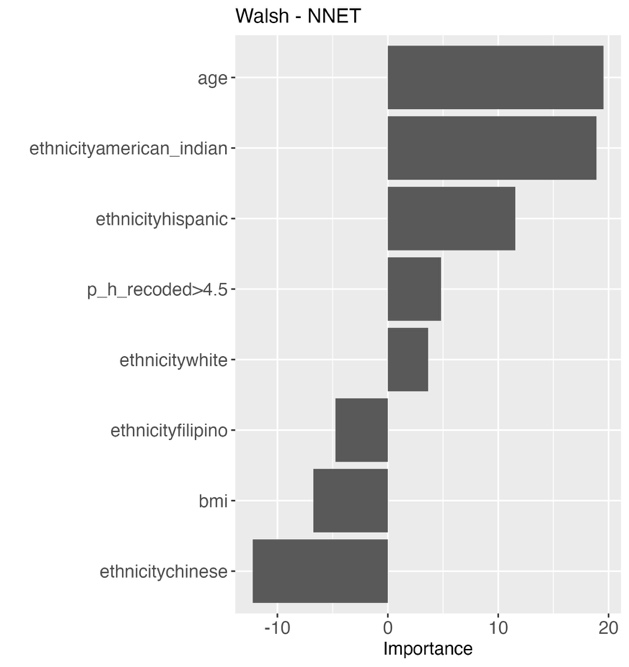


Variable importance for participant characteristics models (without pH)


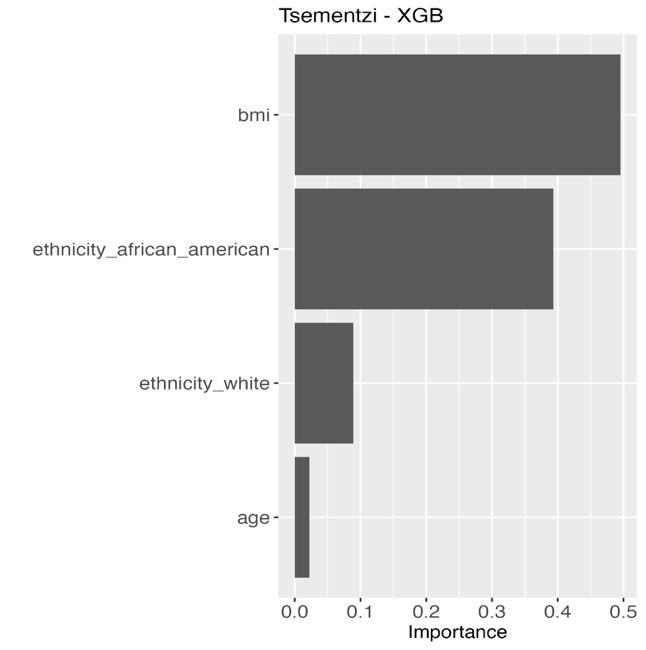

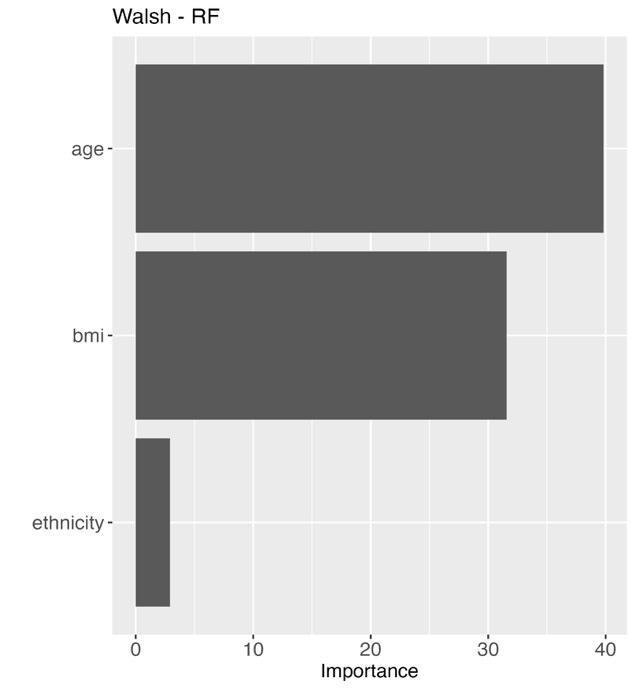

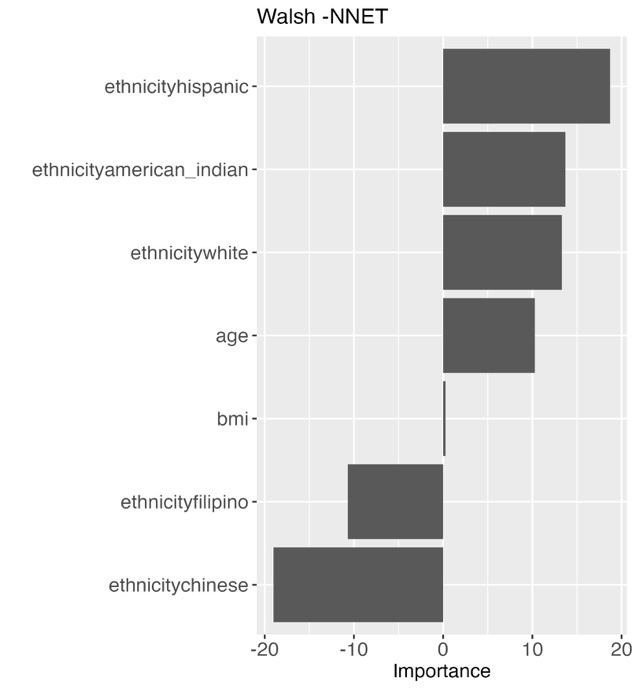


Variable importance for microbiome + participant characteristics + pH


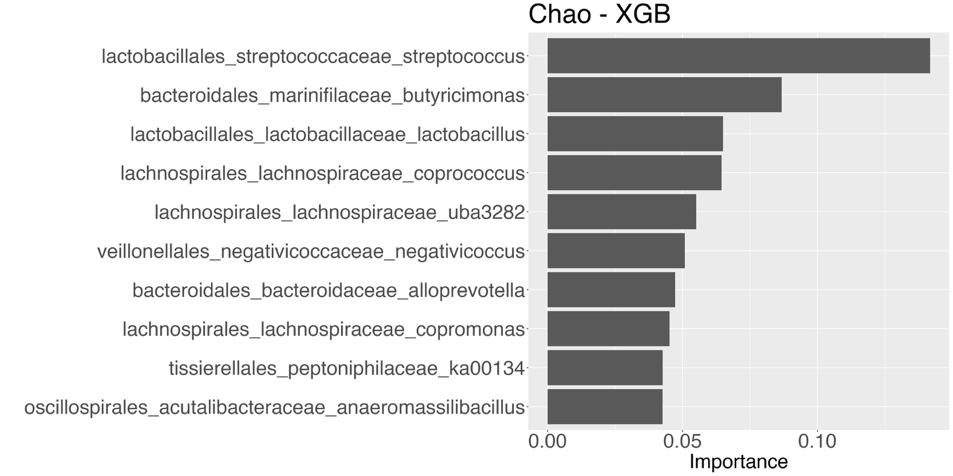

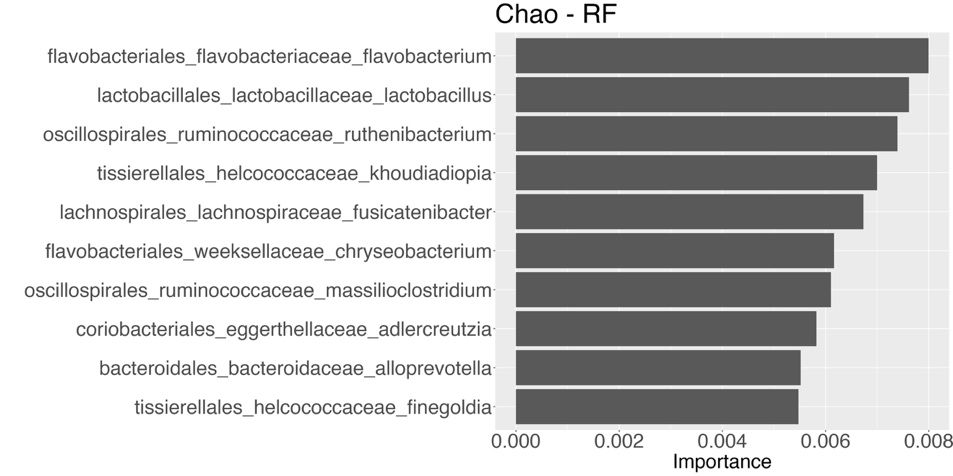

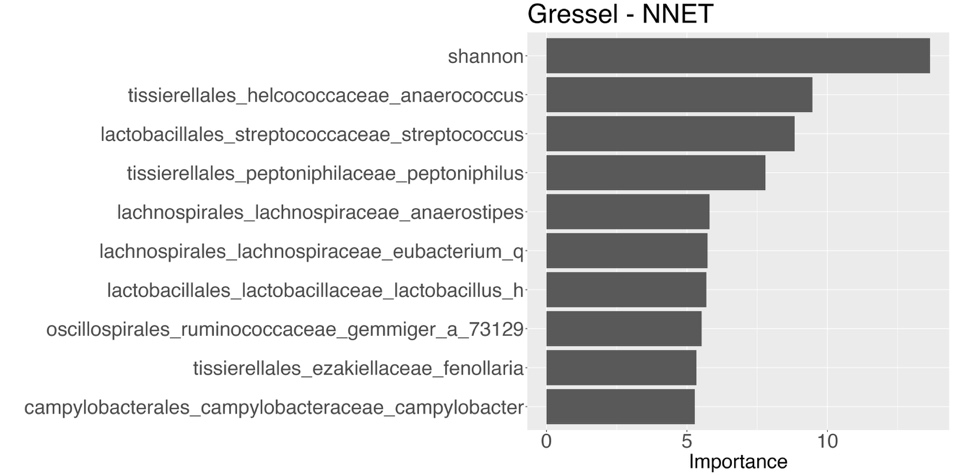

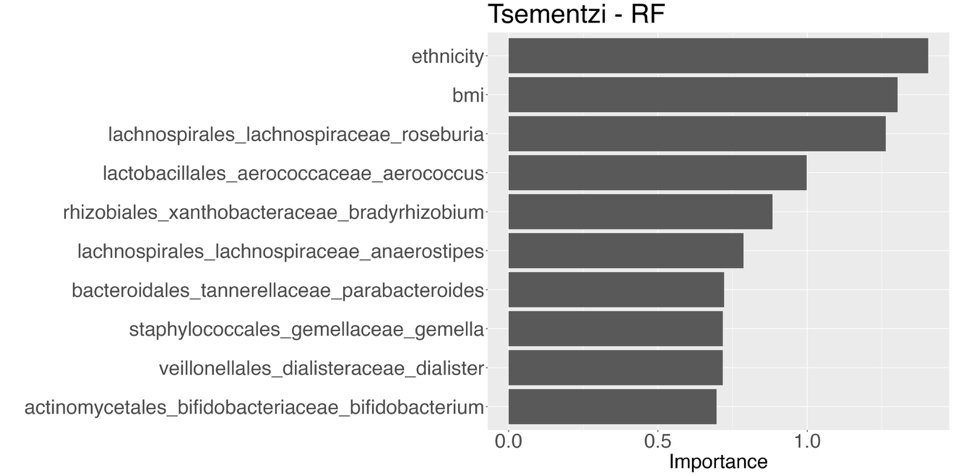

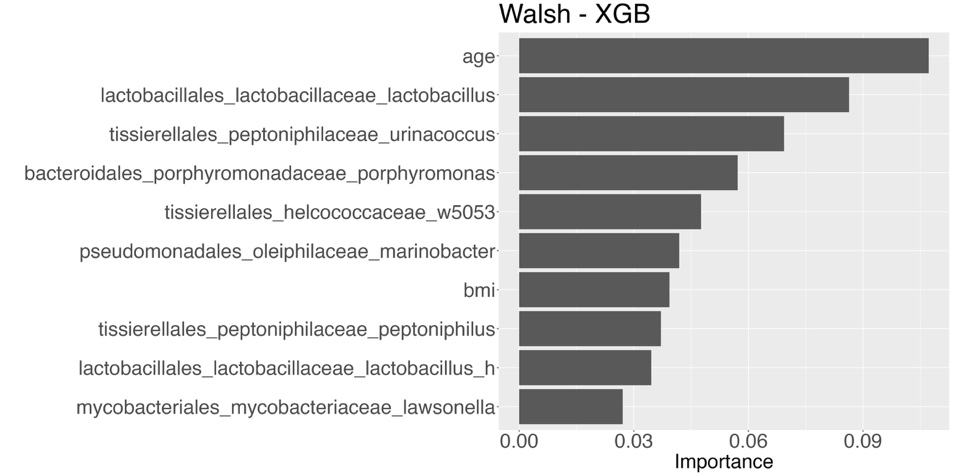

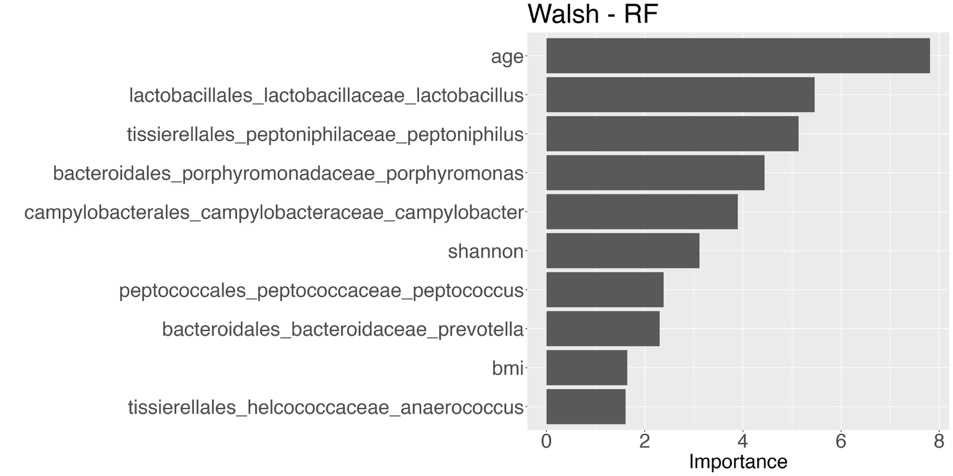


Variable importance for microbiome + participant characteristics without pH


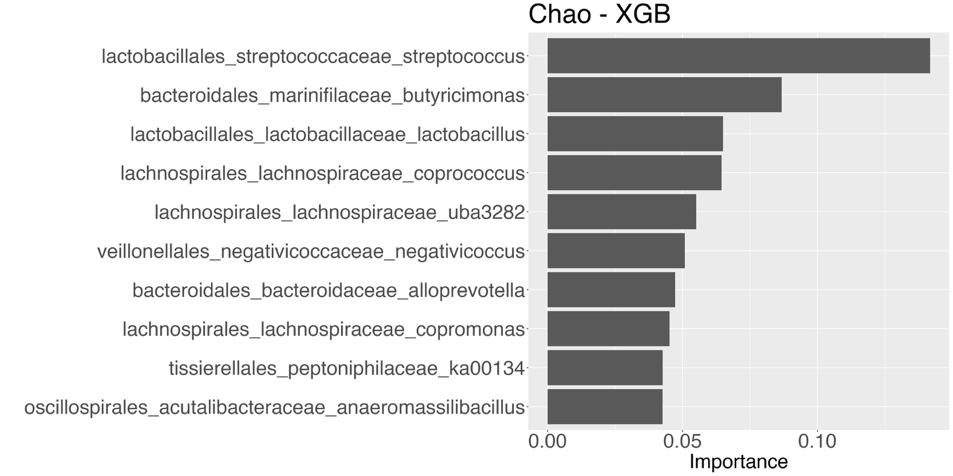

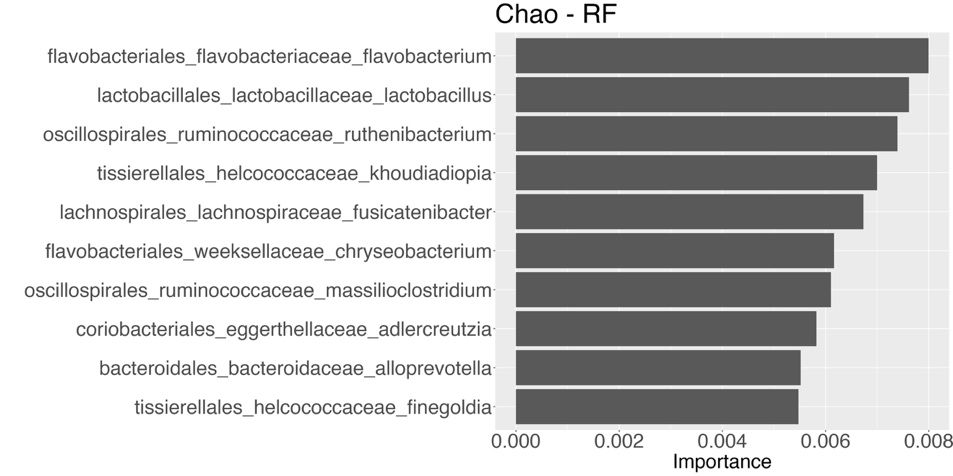

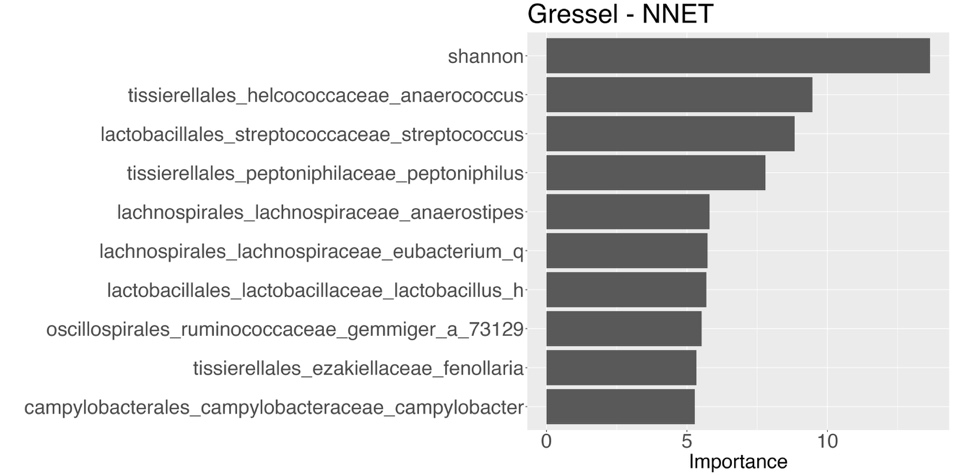

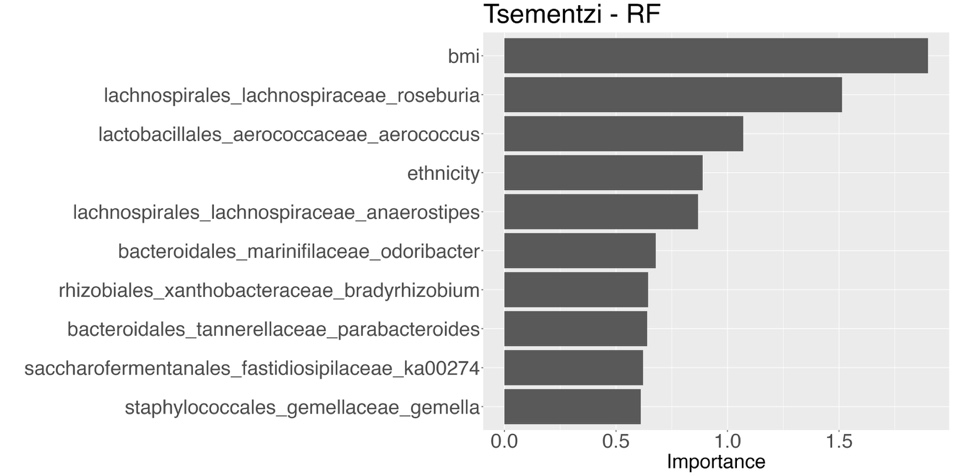

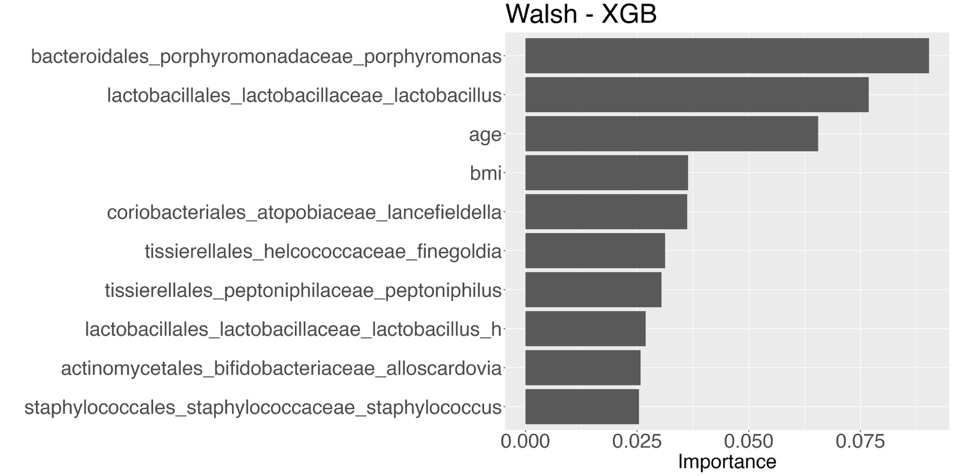

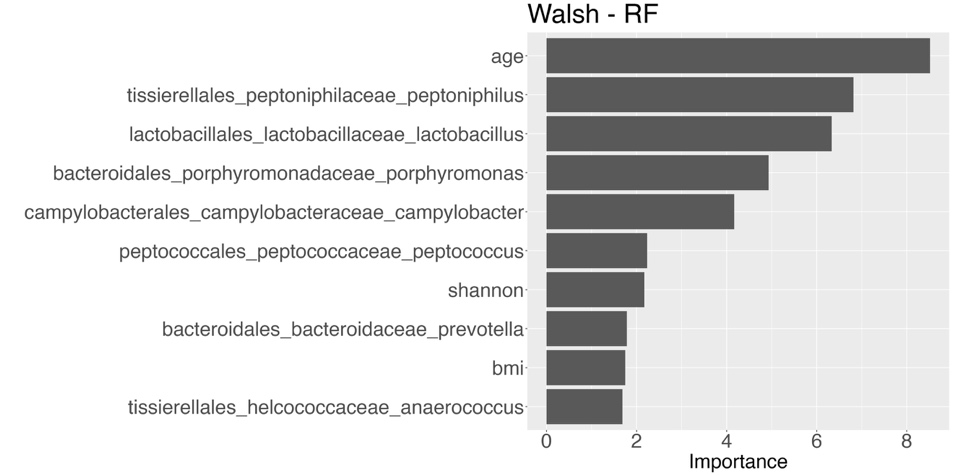


**Section 12: Leave-one-study-out (LOSO) validation results**

Performance metrics with associated 95% confidence intervals for best performing framework LOSO validation. Models were retrained to only use features present in the held-out dataset.

| Held-out dataset (N) | NPV | Sensitivity | PPV | Specificity | AUROC |  |
| --- | --- | --- | --- | --- | --- | --- |
| Antonio et al. (22) | | 1 [0.6, 1] | 1 [0.7, 1] | 0.8 [0.5, 1] | 0.7 [0.4, 0.9] | 0.9 [0.7, 0.9] |
| Walsh et al. (149) | | 0.8 [0.7, 0.9] | 0.8 [0.7, 00.9] | 0.7 [0.6, 0.8] | 0.7 [0.6, 0.8] | 0.8 [0.7, 0.8] |
| Tsementzi et al. (36) | | 0.9 [0.5,1] | 0.5 [0, 1] | 0 [0, 0.2] | 0.2 [0, 0.4] | 0.5 [0.3, 0.8] |
| Gressel et al. (27) | | 0.6 [0.4, 0.8] | 0.2 [0, 0.5] | 0.5 [0, 0.9] | 0.9 [0.6, 1] | 0.7 [0.4, 1] |
| Chao et al. (21) | | 1 [0.9, 1] | 1 [0.4. 1] | 0.5 [0.2, 0.8] | 0.9 [0.7, 1] | 1 [1, 1] |
| Pooled (265) | | 0.7 [0.7, 0.9] | 0.8 [0.7, 0.9] | 0.6 [0.6, 0.7] | 0.5 [0.4, 0.6] | 0.7 [0.6, 0.7] |

*Abbreviations:* NPV: Negative Predictive Value, PPV: Positive Predictive Value, AUROC: Area Under Receiving Operating Characteristics Curve
